# Synthesis and Evaluation of Anisomelic acid-like Compounds for the Treatment of HPV-Mediated Carcinomas

**DOI:** 10.1038/s41598-019-56410-1

**Published:** 2019-12-30

**Authors:** Rajendran Senthilkumar, Yury Brusentsev, Preethy Paul, Parthiban Marimuthu, Fang Cheng, Patrik C. Eklund, John Elias Eriksson

**Affiliations:** 1 Johan Gadolin Process Chemistry Centre, Åbo Akademi University, c/o Laboratory of Organic Chemistry, Biskopsgatan 8, 20500 Turku, Finland; 2 Cell Biology, Faculty of Science and Engineering, Åbo Akademi University, Tykistökatu 6A, 20520 Turku, Finland; 3 Turku Centre for Biotechnology, Åbo Akademi University and University of Turku, Tykistökatu 6A, 20520 Turku, Finland; 4 Structural Bioinformatics Laboratory, Biochemistry, Faculty of Science and Engineering, Åbo Akademi University, Tykistökatu 6A, 20520 Turku, Finland; 5 School of pharmaceutical sciences (Shenzhen), Sun Yat-sen University, 518107 Shenzhen, China

**Keywords:** Oncogenes, Drug discovery and development, Lead optimization

## Abstract

The vast majority of cervical and 75% of oropharyngeal carcinomas are triggered by infection with a type of high-risk oncogenic human papillomavirus (HPV). It is well-known that E6 and E7 oncoproteins are critical for viral-induced cancer, and hence, they represent valuable targets for therapeutic intervention in HPV-mediated cancers. Our earlier research on the cembranoid, anisomelic acid (AA) showed that, AA has the potential to induce apoptosis in HPV cells by the depletion of E6 and E7 oncoproteins. The present study describes the structure-activity relationship and the evaluation of synthetic AA like compounds, i.e simplified cembranoid-like structures, as HPV inhibitors against some papilloma cell lines. Both from experimental and computational results, we observed that these compounds induced apoptosis by the same E6/E7-based mechanism as AA, but at earlier time points, thus being far more effective than AA. Further, the data indicated that only part of the structure of AA is required for the molecular action. Based on these results, we identified some novel and potential compounds for specific treatment of HPV-associated carcinomas.

## Introduction

Human papillomavirus (HPV) is the primary risk factor for cervical cancer^1,2^, the second leading root cause of cancer deaths in women and kills approximately 275, 000 people worldwide each year, and predicted to increase to 474, 000 deaths in 2030^3^. Based on a report from the World Health Organization (2010), it is assessed that the global burden of cervical cancer alone has increased to about 500, 000 new cases annually^3^. Recent studies show that, HPV has been associated with a broad range of other types of epithelial cancers, including oral cancer, various head and neck squamous cell carcinomas (HNSCC) and different forms of anogenital cancers^1,2^, thus, underscoring the importance of the therapeutic development of anti-viral agents against HPV-driven cancers.

The ability of the HPV infection to transform healthy cells into carcinogenic (cancer) cells greatly depends on the two oncoproteins E6 and E7^1^. Both oncoproteins are well-known for their long-term oncogenic potential, and they are present in all HPV genotypes that are mostly responsible for malignant and non-malignant phenotypes. The E6 & E7 protein from the high risk HPV genotypes degrades p53 and cell cycle regulators Rb, p21 and p27 proteins^1,4^. Therefore, the integration of the viral genome and expression of E6 and E7 viral proteins are crucial steps in HPV mediated carcinogenesis. Hence, development of drugs that target HPV E6 and E7 should yield specific and effective treatment of different types of cancers that are dependent on the HPV oncogenes.

Currently, there are no specific treatments for patients with HPV-induced carcinomas. Further, the Food and Drug Administration (FDA) approved drugs specific for the treatment of cervical and HNSCC cancers are only meagre due to their lack of specificity with serious side effects^5^. On the other hand, the existing HPV vaccines cover about seven high-risk HPV types and therefore, they are not appropriate for people who acquire alternative HPV infections from other viral types. The vaccines are also not effective against those who have already been infected with HPV^6^. All of the above aspects constitute an acute need for efficient and specific antiviral HPV-agents.

Anisomelic acid (AA) is one of the principle bioactive compounds isolated from the plant *Anisomeles malabarica*. Recently, we have studied the mode of action of AA-mediated apoptosis, indicating that AA inhibits expression of the E6 and E7 viral proteins^7^. In addition, AA is also effective in inducing apoptosis in other HPV-positive cervical cancer cells, with a high degree of specificity to HPV-positive cells and insignificant effects on HPV-negative cells. This mechanism of action is the first description of AA as a potential inhibitor of oncoprotein expression and as a chemotherapeutic agent for HPV-induced carcinoma. These observations of the antiviral activity of AA^7^ have led us to explore, which type of AA derivatives are more effective in the sensitization of HPV-positive cancer cells to apoptosis, and what are the critical structure determinants and mechanisms of this activity.

The present work aims to identify the most effective compounds from a structure-activity relationship study of AA-like compounds with potent inhibition against HPV E6 and E7 oncoproteins. In order to evaluate the selectivity of the compounds, an *in vitro* high-throughput screening of compounds on various HPV positive genotypes as well as non-cancerous cell lines was conducted. *In silico* docking analysis was utilized in order to identify potential compounds based on docking scores. Further, the direct binding studies of the compounds against E6 and E7 proteins were analyzed using STD NMR. In addition, the preliminary mechanism of action was assessed by flow cytometry and western blot analysis. The *in ovo* chick chorioallantoic membrane (CAM) model was employed to analyze the tumor growth inhibition by the compounds.

## Results and Discussion

The total synthesis of AA (**1**) based on the stereoselective method previously described by Marshall and DeHoff^8^ is time-consuming and expensive. Therefore, we first attempted a computer-aided *in silico* approach to identify potent compounds. This method proved to be largely promising in drug discovery, playing a key role in digging out active leads from large compound libraries^9^.

**Figure 1:**
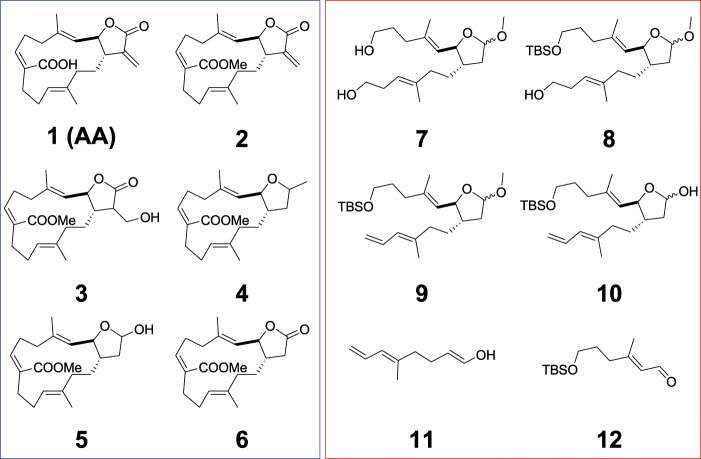
Structure of compounds **1** to **12**.

**Figure 2:**
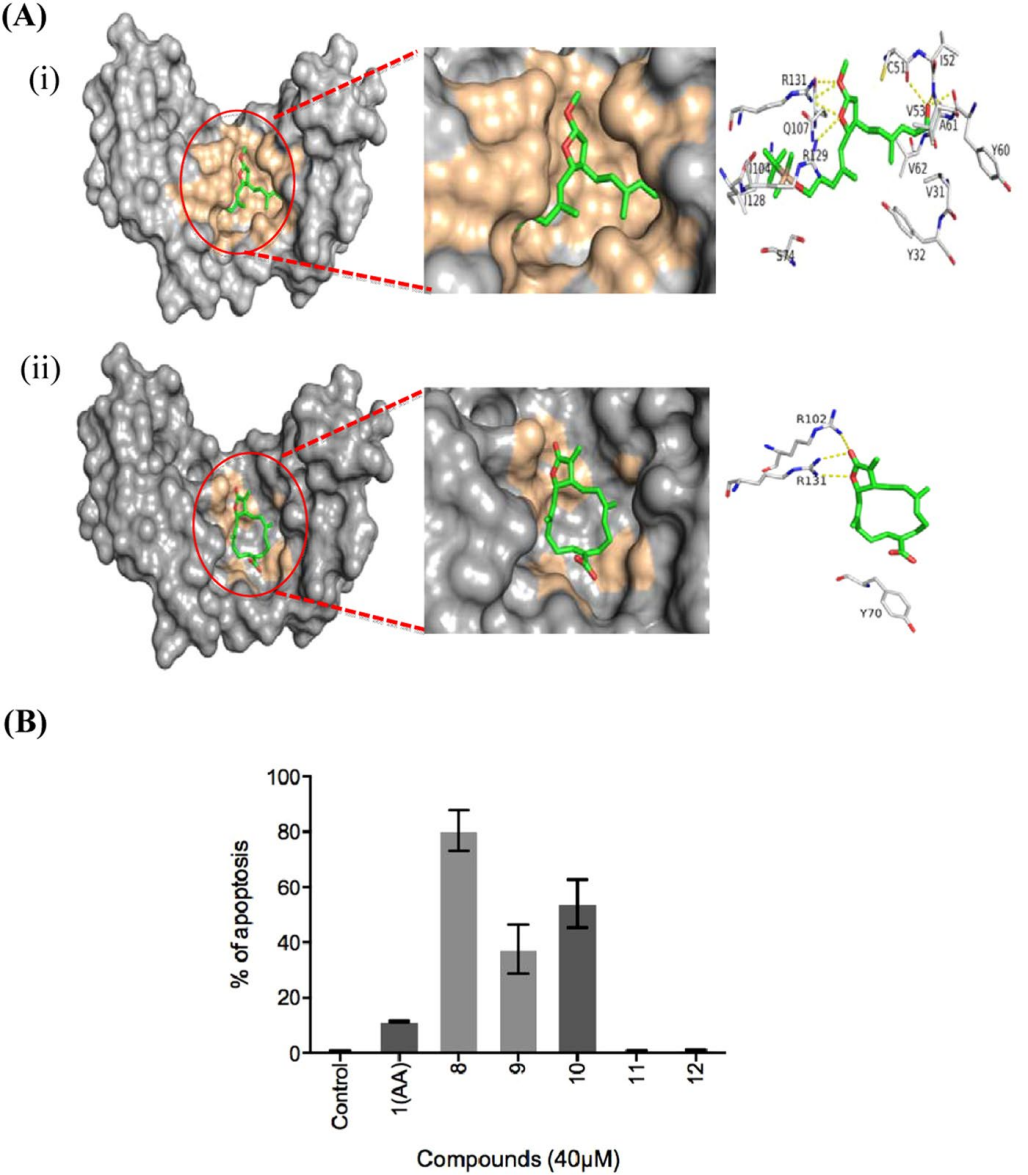
(**A**) Binding modes of compound **8** (i) and anisomelic acid (**1**) (ii), showing the key polar and non-polar interactions predicted obtained using Prime 2.0 and Glide 5.0. (**B**) SiHa cells were subjected to propidium iodide (PI) assay and analyzed by flow cytometry (mean ± SEM; *n* = 3).

The E6 protein is one of the viral oncoproteins that is expressed in HPV-positive cancers and therefore, it was selected as a molecular target for the preliminary *in silico* investigation of a number of compounds involved (**1** to **12**) in the synthesis of AA (**1**) (Fig. 1). The analysis was limited to E6 protein since the crystallographic structure for E7 is not available. On an average, 150–200 conformations were generated per compounds tested. More than 1800 confirmations in total were subjected to the rigid docking filtration approach. The binding site was defined by the crystallographic structure of the E6 protein (PDB ID: 4GIZ). The glide scores obtained by docking of compounds **1**–**12** with E6 are listed in Table 1. The docking scores were generated based on the bonding and nonbonding interactions between the compounds and E6 protein from the Glide scoring function. It was evident from the scores shown in Table 1, that compounds **7** to **10** showed a high glide score. The recently solved x-ray structure of an HPV 16 E6/E6AP complex^10^ revealed that HPV16 E6 formed a distinct binding pocket engaging the ‘LxxLL’ peptide of ubiquitin ligase E6AP, which explains that the pocket is druggable. Interestingly, the open chain compounds (**7** to **10**) were positioned in the hydrophobic cavity of E6 protein (Supplementary Fig. 1), whereas the ring compounds (**1** to **6**) failed to occupy the entire hydrophobic pocket. Figure 2A displays the best binding modes attained for compound **8** and **1** (AA) inside the binding pocket of E6 protein, respectively. Compound **8** was located deep in the hydrophobic cavity surrounded by the following amino acid residues: K11, Y32, F45, D49, L50, C51, V53, A61, V62, L100, I101, R102, A107, W122, G130, and R131. In contrast, compound **1** (AA) failed to establish the proper interaction with the surrounding residues, because the closed ring conformation of AA restricted the docking program from performing an extensive search for the low energy conformational pose inside the E6 binding pocket. This also caused a part of the AA to move outward from the binding pocket of E6 by losing crucial interactions. Due to these effects, the compound **1** only exhibited very weak binding with E6 in comparison with compound **8**.

**Table 1:** *In silico* docking analysis of compounds **1–12**.

Compounds	Docking score (kcal/mol)	Number of H-bonds	Number of salt bridges	Bond length	Interacting residues
**1 (AA)**	−49.49	1	1	3.3	Y70,R102,R131
**2**	−26.85	2	—	2.8	Y32,L50,V62,S74,R129
**3**	−37.64	5	—	2.8	V31,Y32,L50,C51,V53,L67,Y70,S71,Q107,R129
**4**	−26.85	2	—	2.7	V31,Y32,D49,R102,R131
**5**	−27.47	2		3.5	Y32,F45,L67
**6**	−30.40	2	—	2.9	Y32,L50,C51,R129,R131
**7**	−48.01	5	1	3.3	V31,L50,C51,V53,Y60,A61,L67,Q107,R129,R131
**8**	−65.74	5	1	3.6	V31,Y32,C51,I52,V53,Y60,A61,V62,S74,I104,Q107,I128,R129,R131
**9**	−60.65	4	—	3.3	V31,Y32,C51,V53,Y60,A61,V62,L67,S71,S74,Q107,I128,R129
**10**	−63.66	2	1	3.2	V31,Y32,C51,V53,Y60,A61,L67,S74,I104,Q107,I128,R131,T133
**11**	−25.26	2	—	2.6	V31,Y32,F45,L50,C51,V53,Y60,A61,V62,L67
**12**	−24.47	3	—	3.0	Y70,S71,S74,E75,I104,Q107,I128,R129,R131,T133

**Figure 3:**
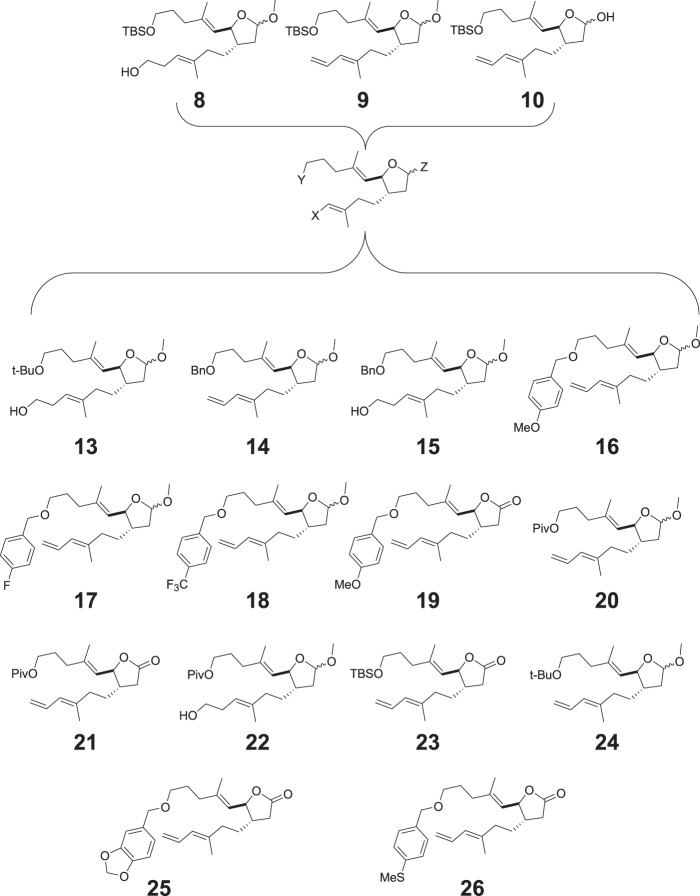
Synthesized compounds with different functionalities at the x, y, z position of a generalized formula.

**Figure 4:**
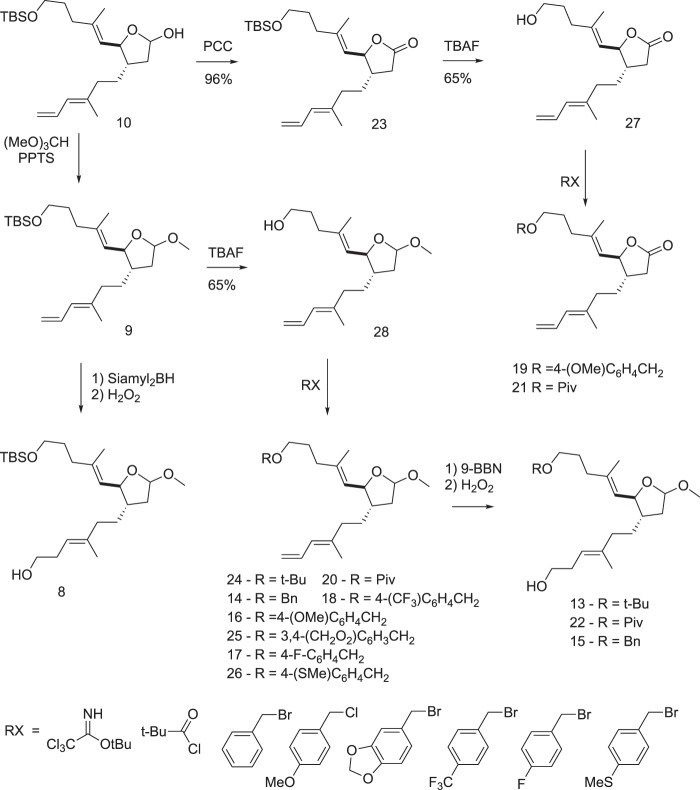
Synthetic route for the compounds **13** to **26**.

Based on the predicted binding modes of AA derivatives to E6 protein, we synthesized compounds **7** to **12**. Further, these compounds were screened for their ability to induce apoptosis in HPV16 cervical cancer cell line, SiHa by caspase 3 staining by flow cytometry analysis (Fig. 2B). The plant-derived compound, AA (**1**) was used as a reference. As illustrated in Fig. 2B, the synthesized compounds **8** to **10** were more efficient in inducing apoptotic cell death in SiHa cell line than AA (**1**). This allowed us to further modify the structure of the compounds **8** to **10** by synthesizing various compounds (**13** to **26**) of different size and hydrophobicity. The varying properties of substituents in the characteristic positions provided us with structure-activity relationship information. In this manner, we were able to construct a generalized formula (see Fig. 3) that can be used for further optimization of the compounds. Variation of X substituent was limited by vinyl and 2-hydroxyethyl groups because of the design of the synthesis. The OH, OMe, OEt and carbonyl groups were used as Z substituent. The following groups: tBuO, MeO, PivO, TBSO, and a number of benzyl-oxy groups were placed to the position of Y substituent. Figure 4 shows the synthetic route of compounds **13** to **26** derived from the generalized formula.

Compounds **8**–**12** were synthesized according to the method described by Marshall *et al*.^8^ Compounds **13–26** were derived either from **10** by oxidation of the lactol to a lactone (**23**) followed by changing the TBS group to the other substituents (**19, 21**) or from **9** by changing the TBS group to the other substituents (**14, 16–18, 20, 24–26**) followed by hydroboration and oxidation of the terminal double bond (**13, 15, 22**) (Fig. 4).

All of the synthesized compounds were examined for their cytotoxicity by the MTT assay on various HPV-positive cervical, patient-derived head and neck cancer cells, as well as HPV-negative normal fibroblast cell lines. The MTT assay was conducted as an indirect measure to evaluate the viability of cells treated with the compounds. Table 2 shows the IC_50_ value of all of the synthesized compounds derived from the generalized formula. It was obvious that most of the compounds showed some selectivity in killing the HPV positive cells compared to the non-cancerous primary skin fibroblast. In particular, compounds **8** and **10** showed higher cytotoxicity at a low IC_50_ dose compared with the other compounds in killing HPV positive cells at the same time showing a higher IC_50_ in non-HPV primary fibroblasts. However, these compounds also were less toxic to primary fibroblast cells when compared to cisplatin and bleomycin (Table 2), which are commonly used drugs in cervical and head and neck cancer treatment.

**Table 2:** *In vitro* screening of the synthesized and benchmark compounds in various HPV genotypes. Values expressed as IC_50_ in μM.

Compounds	SiHa (HPV+)	HeLa (HPV+)	UT-SCC 60 A (HPV+)	UT-SCC 60B (HPV+)	UT-SCC 65 (HPV+)	UT-SCC 69 (HPV+)	K87 (HPV−)	K74 (HPV−)
**13**	—	—	—	45.40	41.18	35.66	—	—
**14**	—	—	48.42	40.73	—	40.13	—	—
**15**	—	51.76	35.99	45.89	40.67	33.50	—	—
**16**	—	50.84	32.89	34.43	—	36.87	40.18	38.82
**17**	—	53.08	45.10	38.73	41.26	30.47	—	—
**18**	39.61	38.40	31.03	39.88	41.89	33.25	—	—
**19**	—	—	—	41.52	42.24	39.43	—	42.30
**20**	39.61	63.88	33.38	—	—	37.60	—	43.03
**21**	—	43.29	27.83	33.21	41.40	39.13	—	38.72
**22**	—	—	29.93	86.50	42.01	35.85	40.63	—
**23**	38.13	62.87	31.42	37.99	42.15	27.73	—	—
**24**	39.60	—	36.72	49.00	—	39.22	—	—
**25**	—	—	62.70	34.17	—	34.44	55.35	—
**26**	39.82	52.48	40.34	47.60	41.44	32.05	—	—
**10**	17.81	22.02	25.21	22.39	24.03	19.19	39.04	37.30
**9**	—	—	44.07	39.65	44.64	37.97	46.66	—
**8**	20.16	22.71	23.25	24.45	23.33	12.73	40.16	46.18
**1 (AA)**	32.08	33.25	17.96	22.82	42.79	23.94	—	—
**Cisplatin**	19.5	—	—	—	—	—	12.17	12.0
**Bleomycin**	22.23	—	—	—	—	—	7.91	8.77

**Figure 5:**
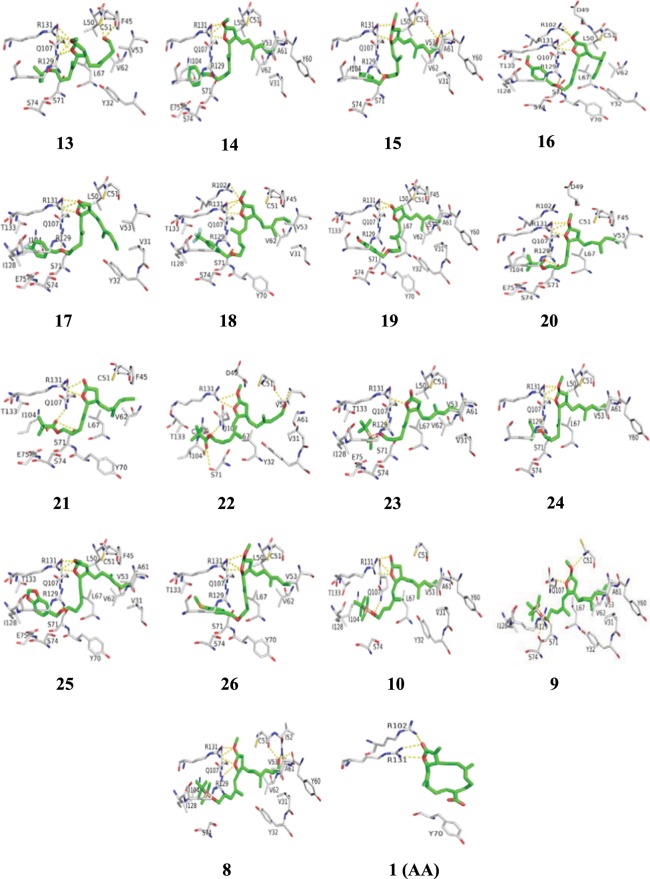
Interaction pattern of Compounds **1, 8–10, 13–26** with E6 protein. The polar interactions are displayed in yellow dotted lines.

In order to understand the plausible binding mode of the synthesized compounds (**8** to **10** and **13** to **26**) inside the hydrophobic pocket of E6, the next round of molecular docking calculations was performed. Interestingly, it was observed that all of the compounds (**8** to **10** and **13** to **26**) properly occupied the hydrophobic binding pocket of E6 in the similar orientation with only a slight difference in the interaction pattern (Fig. 5). Specifically, the number of interacting residues in contact with the ligand varies among the compounds (Supplementary Table 1). As shown in Fig. 5, the residue R131 was strongly interacting with the lactol ring via electrostatic interactions in the majority of cases, and by means of hydrogen bonding in some instances. Also, interactions were established by stable hydrogen bond interactions between residues Q107 and R129 with the polar atoms of the compounds, respectively (Supplementary Table 1). In addition to the graphical investigations, the docking scores obtained from the glide program were also used to rank the binding strength of the synthesized compounds. The resulting docking scores were as high as −65.74 kcal/mol and −63.66 kcal/mol for compounds **8** and **10**, respectively. Therefore, compounds **8** and **10** alone were selected as strong E6 binders, and further studies were carried out only with these two compounds.

**Figure 6:**
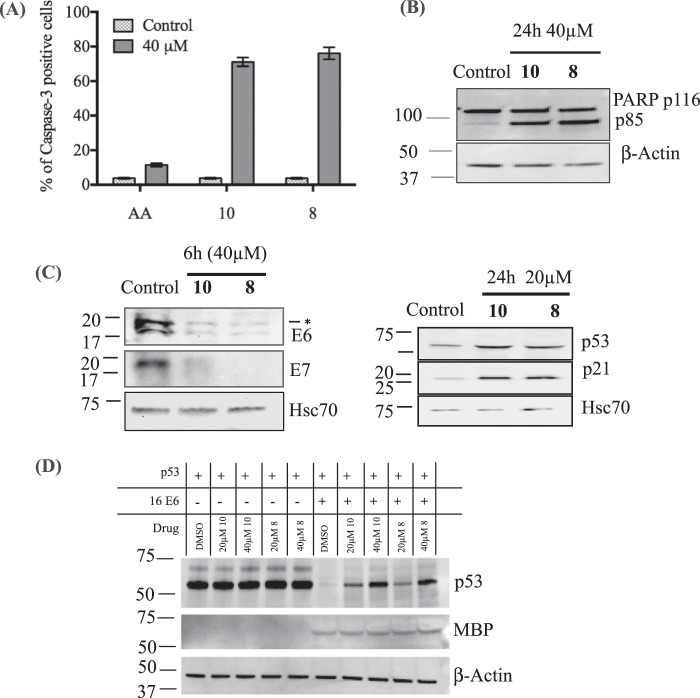
(**A**) Induction of caspase 3 activation by **10** and **8** (mean ± SEM; *n* = 3). (**B)** Western blotting for the expression and cleavage of PARP (116/85). (**C**) Downregulation of E6 (*- unspecific band) and E7 as early as 6 h at 40 µM and upregulation of p53 and p21 at 20 µM at 24 h. (**D**) Representative western blots of p53 and MBP-E6 in the p53 degradation reaction treated with 20 and 40 μM of compounds **10** and **8**. All the gels were run separately and reprobed with either β-Actin or Hsc70 as loading control (The full-length blots are shown in Suppl. Fig. 3).

Subsequently, in order to determine the capacity of the compounds selected from the docking studies to sensitize HPV-driven cervical cancer cell line, SiHa to apoptosis, compounds **8** and **10** were further subjected to the caspase-3 activation assay. A clear activation of caspase-3 (70–80%) was observed after 24 h at 40 μM (Fig. 6A) that confirmed apoptotic cell death induced by the compounds. In order to confirm the apoptosis by Western blotting, lysates of compounds **8** and **10**-treated SiHa cells were analyzed for cleavage of poly-ADP-ribose polymerase (PARP). Treatment with the compounds induced the cleavage of PARP, which is a substrate of caspase-3 (Fig. 6B). In order to further study the effect of the compounds on HPV oncoproteins, E6 and E7, and cell cycle regulators p53 and p21, we treated the cells for 6 h at 40 μM and then collected the cell lysates to analyze the protein expression through Western blot. We found that both compounds downregulated the viral oncoproteins, E6 and E7 and stabilized the cell cycle regulators p53 and p21 respectively (Fig. 6C). The compounds directly or indirectly inhibited the binding of the E6 protein to p53, thereby preventing p53 degradation leading to its stabilization. To further confirm if the compounds could inhibit the ability of E6 to mediate p53 degradation, we assayed compounds, **8** and **10** in a p53 degradation assay. *In vitro* expressed p53 was incubated in rabbit reticulocyte lysate in the presence or absence of *in vitro* expressed HPV16 E6. As shown in Fig. 6D, p53 levels were not reduced in the reaction which did not contain the E6 protein. But, when E6 was added to the DMSO control, p53 was absent showing that it had been degraded by E6. On the other hand, in the reaction that had E6, p53 and the compounds at 2 different concentration (20 and 40 µM), it could be observed that p53 was gradually restored and stabilized. There was no change in the E6 expression. This demonstrates that the compounds are able to inhibit the p53 degradation by E6.

**Figure 7:**
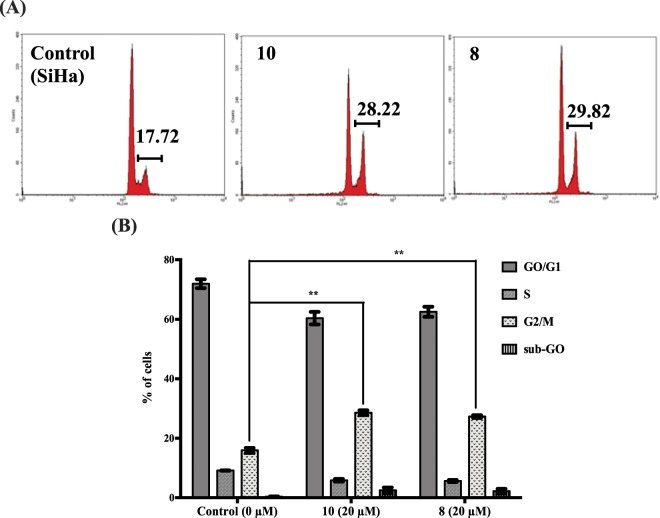
Induction of cell cycle arrest induced by **10** and **8** in HPV positive SiHa cells. (**A**) G2/M cell cycle arrest at 20 µM at 24 h. (**B**) Graph showing the percentage of G2/M cell cycle arrest (mean ± SEM; *n* = 3; **P ≤ 0.01 (t test)).

We also found that p21 was upregulated at the lower dose (20 µM), and p21 is one of the downstream targets of p53^11^. Further, the cell cycle assay results (Fig. 7) showed that there was a significant increase in the accumulation of cells in the G2/M phase, which led to G2/M cell cycle arrest, which in turn occurred due to the stabilization of the cell cycle regulators like p21. Many studies have shown that, p21 can be activated *via* different upstream responses and p53 is one among them^11^. In the case of AA, we have previously shown that when p53 stabilization was inhibited by p53 shRNA, p21 did not get stabilized and there was no G2/M arrest, which demonstrates that p53 activates p21^7^. It is also well known that the carboxyl termini of the high-risk E7 proteins bind p21 efficiently and neutralize its inhibitory effect^12^. Further, that it is indispensable that this inhibitory effect of E7 on p21 has to be eliminated for p21 to be activated. Thus, we hypothesize that these compounds also follow the similar mechanism of action like AA and, therefore, both the combined effect of E7 down-regulation and p53 stabilization, simultaneously lead to the activation of p21 thus leading to G2/M arrest.

In order to study the direct binding of the compounds **8** and **10** to oncoproteins E6 and E7, we performed a saturation transfer difference NMR study (STD-NMR), according to previously published procedures^13^. STD-NMR spectra showed hydrophobic interactions between compounds **8** and **10** with the proteins (Suppl. Fig. 2). However, because of the lack of the sufficient amount of proteins, we were not able to determine the binding constant. Therefore, more insight into the direct binding mechanism warrants further studies.

**Figure 8:**
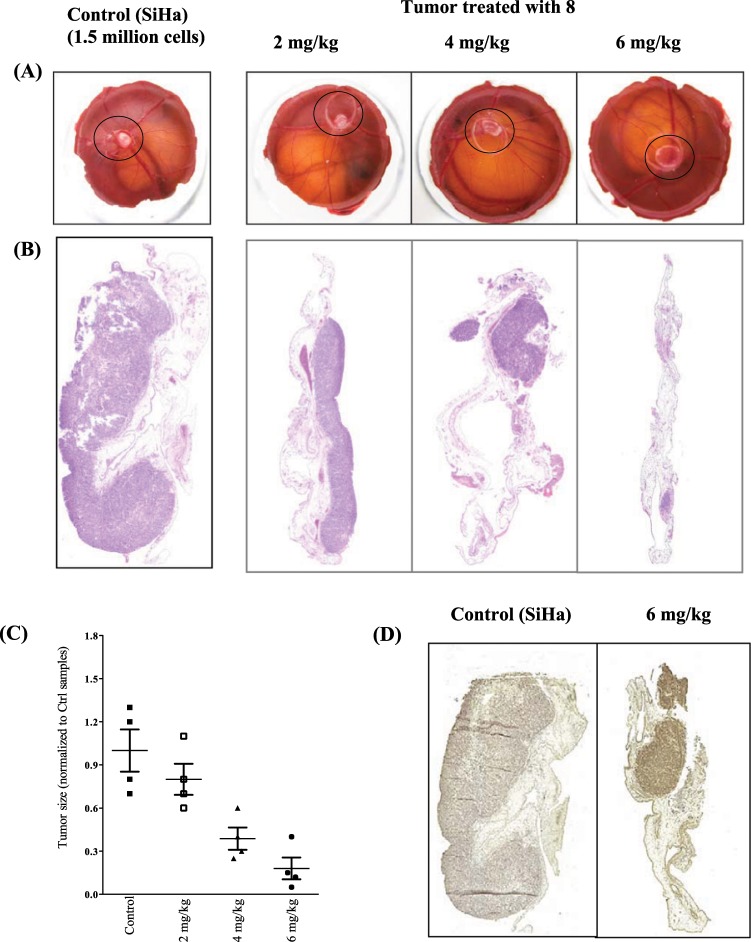
(**A**) Inhibition of growth in SiHa cells in the CAM *in ovo* tumor growth model, by compound **8**; the tumor inside the plastic ring on the CAM are marked (**B**) Representative pictures of H and E staining of SiHa tumor cells in the CAM *in ovo* tumor growth model upon different concentration of treatment (mean ± SEM; *n* = 4); (**C**) Quantification of SiHa tumor size indicated in panel. (**D**) Immunohistochemical labeling of Bad antibody showing the increase of apoptosis in the tumor cells after treatment with compound **8**.

The chicken chorioallantoic membrane (CAM) test system represents an intermediate state between *in vitro and in vivo* systems. Chick embryos can be employed to examine the inhibition activity or toxicity of a compound on the CAM and/or CAM-grafted tumors^14^. For these reasons, we employed the CAM model to study the preliminary *in vivo* efficacy of one of the lead compounds - compound **8**. The tumor inhibition efficacy of the compound was tested by the topical treatment on cervical tumors (SiHa) grown on CAM. The tumours were treated with the equivalent of 2 mg/kg, 4 mg/kg and 6 mg/kg of **8** (Fig. 8). It was observed that the cervical cancer cell, SiHa, had formed a prominent tumor in the CAM model and that already a dose range of 4 mg/kg of **8** had inhibited the CAM tumor growth. Moreover, a dose range of 6 mg/kg was able to diminish the already formed tumor (Fig. 8A,C). The efficacy of **8** on CAM tumor growth inhibition was also confirmed by hematoxylin/eosin (H and E) staining, as well as by the immunohistochemistry methods using cell proliferation and apoptotic markers (Fig. 8B,D). As depicted in Fig. 8, the illustrative control section showed solidly populated tumor cells all over the CAM tissue, while at a dosage of 2 mg/ml of **8**, the regression of tumor growth was becoming evident. The effect of compound **8** was in dose-dependent modus at 4 mg/ml, the CAM tumor size was notably smaller than the control sample; and at 6 mg/ml, the CAM tumor was almost completely regressed. The quantitative data are shown in Fig. 8C. The induction of apoptosis by compound **8** was confirmed by staining of Bad antibody in the *in ovo* CAM model (Fig. 8D).

Conclusively, the current investigation demonstrated the obvious applicability and the structure-activity relationship of synthetic AA like compounds. The *in-silico* docking technique predicted the low energy conformation poses, revealed the crucial residues involved in the interactions with the surrounding residues and also ranked the compounds based on the binding scores. The compounds selected from the docking results were further evaluated for their efficacy and selectivity towards HPV positive and normal primary fibroblast cell lines. Our results clearly exhibited that compounds **8** and **10** were highly effective with low IC_50_ compared to AA and other compounds in the series. Moreover, those compounds also exhibited less toxicity to primary fibroblasts compared to the commercial drugs. Although we observed higher potencies for these selected compounds than AA, our data suggest that further derivatization and optimization of these compounds could potentially lead to a synthesis of novel compounds with the high potency for the treatment of HPV-mediated cancers.

## Experimental Section

### General information

Chemicals were purchased from commercial suppliers and used as such. 4-methoxybenzyl bromide was prepared from 4-methoxybenzyl alcohol (reaction with hydrobromic acid in water solution followed by separation and drying of the product) a few days prior use and stored with calcium carbonate as a stabilizer. THF was dried by the sodium-benzophenone method immediately before use.

NMR spectra were recorded with Bruker Avance 600 MHz and Bruker Avance 500 MHz NMR spectrometers using standard pulse sequences. NMR-data is reported only for novel compounds. HRMS were recorded using a Bruker Micro Q-TOF instrument with ESI (electrospray ionization) operated in positive or negative mode. The reactions were monitored by TLC method. Aluminum based TLC plates (Merck) silica gel 60 F_254_ were used.

### Synthesis of compounds

Compounds **8–10** were prepared by the method described by Marshall *et al*.^8^.

#### Preparation of the lactone 23

**10 (**100 mg, 0,25 mmol) was dissolved in DCM (5 ml) and pyridinium chlorochromate (93 mg, 0,43 mmol) was added in one portion. The reaction mixture was stirred for 3 h and then diluted by diethyl ether (10 ml). The formed suspension was filtered through celite pad and the filtrate was concentrated to dryness. The residue was finally purified by flash chromatography on silica gel in CHCl_3_/Ethanol 20:1 to get lactone **23** (96 mg, 0.24 mmol, 96% yield) as colorless viscous oil. ^1^H NMR (500 MHz, CHLOROFORM-*d*) δ ppm 0.05 (s, 6 H) 0.90 (s, 9 H) 1.37–1.55 (m, 1 H) 1.56–1.72 (m, 3 H) 1.74 (s, 3 H) 1.76 (d, *J* = 0.92 Hz, 3 H) 1.98–2.19 (m, 5 H) 2.23 (dd, *J* = 16.63, 10.68 Hz, 1 H) 2.69 (dd, *J* = 16.71, 7.55 Hz, 1 H) 3.61 (t, *J* = 6.33 Hz, 2 H) 4.79 (t, *J* = 8.62 Hz, 1 H) 5.03 (dd, *J* = 10.07, 0.92 Hz, 1 H) 5.12 (dd, *J* = 16.78, 1.00 Hz, 1 H) 5.21 (dd, *J* = 8.93, 0.99 Hz, 1 H) 5.83 (d, *J* = 10.83 Hz, 1 H) 6.55 (dt, *J* = 16.78, 10.53 Hz, 1 H). ^13^C NMR (126 MHz, CHLOROFORM-*d*) δ ppm −5.29, 16.42, 17.08, 18.32, 25.94, 29.99, 30.74, 35.48, 35.79, 37.76, 42.72, 62.46, 82.21, 115.59, 121.73, 126.33, 132.92, 137.64, 144.20, 176.33. HRMS (ESI): calcd. for C_23_H_40_NaO_3_Si [M + Na]^+^ 415.2639; found 415.2641.

#### Preparation of the alcohols 27 and 28

**23** (470 mg, 1,2 mmol) or **9** (400 mg, 0,98 mmol) was dissolved in 5 ml of THF and tetrabutylammonium fluoride trihydrate (620 mg, 2 mmol) was added in one portion. The mixture was stirred at room temperature for 3 h and then diluted with 10 ml of saturated ammonium chloride solution and 3 ml of water. The reaction mixture was extracted three times with EtOAC (3 × 10 ml). The combined organic phases were concentrated to dryness and the residue was purified by column chromatography on silica (eluent CHCl_3_ to CHCl_3_/Acetone 30:1) to give the product **27** (236 mg, 0.86 mmol, 71%) or **28** (190 mg, 0.64 mmol, 65%) as colorless viscous oils.

#### Preparation of the tBu-ether 24

**28** (53 mg, 0,180 mmol) was dissolved in a mixture of 1 ml cyclohexane and 0,3 ml of DCM^15^. Tert-butyl 2,2,2-trichloroacetimidate (118 mg, 0.540 mmol) was added in one portion followed by addition of catalytic amount of boron trifluoride etherate (4,46 µl, 0.035 mmol). The mixture was stirred overnight (16 h) at room temperature and solid NaHCO_3_ (20 mg) was added. After stirring additional 15 min the mixture was filtrated through a silica gel pad and the filtrate was concentrated to dryness. The residue was purified by column chromatography on silica (eluent CHCl_3_/Ethanol 20:1) to give the desired tBu ether **24** (21 mg, 0.059 mmol, 33%) and recovered starting material **28** (30 mg, 0.1 mmol, 57%).

#### **24**

^1^H NMR (500 MHz, CHLOROFORM-*d*) δ ppm 1.18 (d, *J* = 2.59 Hz, 10 H) 1.30–1.45 (m, 1 H) 1.47–1.69 (m, 5 H) 1.70–1.77 (m, 6 H) 1.96–2.17 (m, 5 H) 2.35–2.46 (m, 1 H) 3.18–3.38 (m, 5 H) 4.34 (t, *J* = 8.93 Hz, 1 H) 4.88–5.02 (m, 2 H) 5.05–5.22 (m, 2 H) 5.82 (d, *J* = 10.83 Hz, 1 H) 6.46–6.59 (m, 1 H); ^13^C NMR (126 MHz, CHLOROFORM-*d*) δ ppm 16.49, 16.54, 16.57, 16.99, 27.59, 28.57, 28.63, 30.01, 30.42, 36.21, 36.28, 38.57, 38.58, 39.52, 39.97, 43.33, 45.12, 54.40, 55.19, 61.09, 61.14, 72.52, 78.92, 82.04, 104.54, 104.85, 114.84, 114.89, 123.82, 125.61, 125.70, 126.48, 133.26, 139.03, 139.32, 141.39; HRMS (ESI): calcd. for C_22_H_38_NaO_3_ [M + Na]^+^ 373.2713; found 373.2721.

#### Preparation of the pivaloyl esters 21 and 20

**27** (93 mg, 0,34 mmol) or **28** (100 mg, 0,34 mmol) was dissolved in 1 ml of DCM. Triethylamine (0,066 ml, 0,48 mmol) was added followed by addition of pivaloyl chloride (0,050 ml, 0,41 mmol) and DMAP (4 mg, 0,034 mmol) The mixture was stirred 3 h at room temperature and then diluted with 3 ml of DCM. The solution was extracted with water (2 × 4 ml), washed with brine (4 ml), dried over sodium sulfate and concentrated to dryness. The residue was purified by column chromatography on silica (eluent EtOAc/Hexane 1:20) to give the product **21** (96 mg, 0.27 mmol, 80% yield) or **20** (108 mg, 0.29 mmol, 84% yield) as a colorless solids.

#### **21**

^1^H NMR (500 MHz, CHLOROFORM-*d*) δ ppm 1.20 (s, 9 H) 1.77 (s, 4 H) 1.74 (s, 3 H) 1.77 (s, 3 H) 2.01–2.33 (m, 5 H) 2.23 (dd, *J* = 16.78, 10.68 Hz, 1 H) 2.70 (dd, *J* = 16.78, 7.63 Hz, 1 H) 3.99–4.09 (m, 2 H) 4.32–4.57 (m, 1 H) 4.78 (t, *J* = 8.54 Hz, 1 H) 5.02 (dd, *J* = 10.15, 1.14 Hz, 1 H) 5.11 (dd, *J* = 16.78, 1.37 Hz, 1 H) 5.22 (dd, *J* = 8.93, 1.14 Hz, 1 H) 5.83 (d, *J* = 10.68 Hz, 1 H) 6.55 (dt, *J* = 16.78, 10.53 Hz, 1 H); ^13^C NMR (126 MHz, CHLOROFORM-*d*) δ ppm 16.42, 17.01, 26.64, 27.21, 29.99, 35.42, 35.76, 37.75, 38.76, 42.70, 63.54, 82.02, 115.61, 122.40, 126.35, 132.93, 137.63, 143.16, 176.27, 178.49; HRMS (ESI): calcd. for C_22_H_34_NaO_4_ [M + Na]^+^ 385.2349; found 385.2355.

#### **20**

^1^H NMR (500 MHz, CHLOROFORM-*d*) δ ppm 1.17–1.22 (m, 9 H) 1.31–1.68 (m, 4 H) 1.70–1.76 (m, 7 H) 1.93–2.47 (m, 6 H) 3.32–3.39 (m, 3 H) 4.02–4.11 (m, 2 H) 4.27–4.37 (m, 1 H) 4.83–5.03 (m, 2 H) 5.09 (d, *J* = 16.78 Hz, 1 H) 5.14–5.23 (m, 1 H) 5.83 (d, *J* = 10.83 Hz, 1 H) 6.47–6.59 (m, 1 H)**;** ^13^C NMR (126 MHz, CHLOROFORM-*d*) δ ppm 16.47, 16.49, 16.57, 16.97, 26.77, 27.08, 27.22, 29.97, 30.41, 35.84, 35.89, 38.49, 38.75, 39.49, 39.94, 43.27, 45.04, 54.44, 55.20, 63.78, 63.84, 78.83, 81.90, 104.63, 104.89, 114.86, 114.90, 124.46, 125.60, 125.69, 127.17, 133.25, 138.35, 139.01, 140.40, 178.51; HRMS (ESI): calcd. for C_23_H_38_NaO_4_ [M + Na]^+^ 401.2662; found 401.2671.

#### General procedure for preparation of the benzyl ethers **14**, **16**, **17**, **18**, **19**, **25** and **26**

Starting alcohol (1 equiv. of **27** or 1 equiv. of **28**) was dissolved in DMF (1 ml per 0,3 mmol of **27** or **28**) and cooled down to −20 °C. Li-HMDS was slowly added and the solution was stirred for 10 min. The corresponding benzyl bromide (or chloride) was added dropwise then cooling was removed and the mixture was stirred for 4 h at room temperature. Then, the mixture was diluted with 3 ml of water and extracted with diethyl ether (2 × 4 ml). The combined ether extracts were washed with water (2 × 5 ml) and with brine (4 ml) and dried over sodium sulfate. The solvent was then concentrated and the residue was purified by column chromatography (EtOAc/Hexane 1:20) to give the resulting product **14, 16, 17, 18, 19, 25** and **26**.

**14** (223 mg, 86% yield). ^1^H NMR (500 MHz, CHLOROFORM-*d*) δ ppm 1.23–1.63 (m, 2 H) 1.52–1.66 (m, 1 H) 1.68–2.08 (m, 1 H) 1.70–1.74 (m, 6 H) 1.75–1.83 (m, 2 H) 1.94–2.09 (m, 2 H) 2.08–2.17 (m, 2 H) 2.17–2.49 (m, 1 H) 3.29–3.40 (m, 3 H) 3.41–3.54 (m, 2 H) 4.34 (t, *J* = 8.93 Hz, 1 H) 4.49 (br. s, 2 H) 4.89–5.03 (m, 2 H) 5.08 (d, *J* = 16.78 Hz, 1 H) 5.12–5.24 (m, 1 H) 5.82 (d, *J* = 10.07 Hz, 1 H) 6.55 (ddd, *J* = 16.78, 10.70, 10.10 Hz, 1 H) 7.26–7.31 (m, 1 H) 7.31–7.36 (m, 4 H); ^13^C NMR (126 MHz, CHLOROFORM-*d*) δ ppm 16.49, 16.53, 16.58, 16.99, 27.81, 27.84, 29.98, 30.40, 36.14, 36.19, 38.52, 38.53, 39.51, 39.96, 43.29, 45.05, 54.41, 55.19, 69.96, 72.90, 72.97, 78.89, 81.99, 104.57, 104.87, 114.87, 114.91, 124.03, 125.59, 125.69, 126.69, 127.51, 127.61, 127.63, 128.37 (s, 2 C) 133.25, 138.59, 139.03, 141.11; HRMS (ESI): calcd. for C_25_H_36_NaO_3_ [M + Na]^+^ 407.2557; found 407.2533.

**16** (70 mg, 50% yield). ^1^H NMR (500 MHz, CHLOROFORM-*d*) δ ppm 1.12–2.51 (m, 17 H) 3.24–3.39 (m, 3 H) 3.40–3.49 (m, 2 H) 3.80 (s, 3 H) 4.34 (t, *J* = 8.93 Hz, 1 H) 4.37–4.49 (m, 2 H) 4.84–5.03 (m, 2 H) 5.08 (d, *J* = 16.94 Hz, 1 H) 5.11–5.24 (m, 1 H) 5.82 (d, *J* = 10.83 Hz, 1 H) 6.55 (dt, *J* = 16.86, 10.49 Hz, 1 H) 6.87 (d, *J* = 7.93 Hz, 2 H) 7.26 (dd, *J* = 8.24, 1.68 Hz, 2 H)**;** ^13^C NMR (126 MHz, CHLOROFORM-*d*) δ ppm 16.48, 16.52, 16.57, 16.97, 27.80, 27.82, 29.97, 30.39, 36.14, 36.19, 38.51, 38.53, 39.50, 39.96, 43.28, 45.04, 54.39, 55.17, 55.25, 69.63, 69.66, 72.54, 72.65, 78.89, 81.99, 104.56, 104.86, 113.76, 114.86, 114.91, 124.03, 125.59, 125.67, 126.68, 129.21, 129.23, 130.68, 133.24, 139.01, 139.06, 141.08, 159.12; HRMS (ESI): calcd. for C_26_H_37_NaO_4_ [M + Na]^+^ 437.2662; found 437.2652.

**17** (72 mg, 67.7% yield). ^1^H NMR (500 MHz, CHLOROFORM-*d*) δ ppm 1.27–2.49 (m, 17 H) 3.26–3.41 (m, 3 H) 3.41–3.50 (m, 2 H) 4.29–4.38 (m, 1 H) 4.45 (s, 2 H) 4.89–5.03 (m, 2 H) 5.08 (d, *J* = 16.94 Hz, 1 H) 5.12–5.23 (m, 1 H) 5.82 (d, *J* = 10.38 Hz, 1 H) 6.55 (dt, *J* = 16.90, 10.47 Hz, 1 H) 6.99–7.06 (m, 2 H) 7.30 (ddd, *J* = 8.35, 5.61, 2.37 Hz, 2 H); ^13^C NMR (126 MHz, CHLOROFORM-*d*) δ ppm 16.48, 16.53, 16.58, 16.97, 27.75, 27.77, 29.97, 30.39, 36.10, 36.16, 38.51, 38.54, 39.50, 39.96, 43.29, 45.06, 54.41, 55.20, 69.94, 69.96, 72.19, 72.25, 78.87, 81.98, 104.60, 104.87, 114.89, 114.93, 115.20 (d, *J* = 21.80 Hz, 1 C) 124.11, 125.60, 125.69, 126.77, 129.29 (d, *J* = 1.82 Hz, 1 C) 129.36 (d, *J* = 1.82 Hz, 1 C) 133.24, 134.31, 134.34, 138.94, 139.01, 140.98, 161.30, 163.25; HRMS (ESI): calcd. for C_25_H_35_FNaO_3_ [M + Na]^+^ 425.2462; found 425.2464.

**18** (76 mg, 49.4% yield). ^1^H NMR (500 MHz, CHLOROFORM-*d*) δ ppm 1.34–1.54 (m, 2 H) 1.57–1.65 (m, 1 H) 1.66–1.84 (m, 9 H) 1.95–2.18 (m, 4 H) 2.34–2.47 (m, 1 H) 3.31–3.42 (m, 3 H) 3.45–3.52 (m, 2 H) 4.34 (t, *J* = 8.93 Hz, 1 H) 4.54 (s, 2 H) 4.86–5.02 (m, 2 H) 5.08 (d, *J* = 16.78 Hz, 1 H) 5.13–5.24 (m, 1 H) 5.79–5.84 (m, 1 H) 6.55 (dt, *J* = 16.82, 10.51 Hz, 1 H) 7.45 (d, *J* = 7.63 Hz, 2 H) 7.60 (d, *J* = 7.93 Hz, 2 H)**;** ^13^C NMR (126 MHz, CHLOROFORM-*d*) δ ppm 16.48, 16.53, 16.58, 16.97, 27.73, 27.76, 29.97, 30.38, 36.08, 36.13, 38.51, 38.53, 39.51, 39.95, 43.30, 45.07, 54.42, 55.20, 70.31, 70.33, 72.07, 72.13, 78.86, 81.97, 104.61, 104.88, 114.90, 114.94, 124.19, 125.27, 125.29, 125.32, 125.60, 125.69, 126.85, 127.46, 127.48, 133.24, 138.85, 139.01, 140.90, 142.75; HRMS (ESI): calcd. for C_26_H_34_F_3_NaO_3_ [M + Na]^+^ 475.2431; found 475.2428.

**19** (85 mg, 59% yield). ^1^H NMR (600 MHz, CHLOROFORM-*d*) δ ppm 1.41–1.52 (m, 1 H) 1.61–1.71 (m, 1 H) 1.71–1.74 (m, 2 H) 1.75 (s, 3 H) 1.76 (d, *J* = 1.13 Hz, 3 H) 1.98–2.12 (m, 2 H) 2.16 (t, *J* = 7.37 Hz, 2 H) 2.23 (dd, *J* = 17.00, 10.58 Hz, 1 H) 2.34–2.39 (m, 1 H) 2.70 (dd, *J* = 17.00, 7.93 Hz, 1 H) 3.45 (t, *J* = 6.42 Hz, 2 H) 3.82 (s, 3 H) 4.44 (s, 2 H) 4.79 (t, *J* = 8.69 Hz, 1 H) 5.04 (d, *J* = 10.50 Hz, 1 H) 5.13 (d, *J* = 16.62 Hz, 1 H) 5.19 (d, *J* = 8.69 Hz, 1 H) 5.84 (d, *J* = 10.58 Hz, 1 H) 6.56 (ddd, *J* = 16.60, 10.60, 10.50 Hz, 1 H) 6.90 (ddd, *J* = 8.69, 2.40, 2.00 Hz, 2 H) 7.27 (ddd, *J* = 8.70, 2.40, 2.00 Hz, 2 H); ^13^C NMR (151 MHz, CHLOROFORM-*d*) δ ppm 16.44, 17.03, 27.67, 29.99, 30.19, 30.29, 35.48, 36.04, 55.29, 69.22, 72.63, 82.16, 113.79, 115.63, 121.97, 126.34, 129.29, 130.57, 132.92, 137.65, 143.98, 159.17, 176.35; HRMS (ESI): calcd. for C_25_H_34_NaO_4_ [M + Na]^+^ 421.2349; found 421.2348.

**25** (73 mg, 50.3% yield). ^1^H NMR (500 MHz, CHLOROFORM-*d*) δ ppm 1.23–1.54 (m, 2 H) 1.56–1.64 (m, 1 H) 1.66–1.80 (m, 9 H) 1.96–2.18 (m, 5 H) 2.35–2.46 (m, 1 H) 3.31–3.46 (m, 5 H) 4.32–4.42 (m, 3 H) 4.91–5.03 (m, 2 H) 5.05–5.22 (m, 2 H) 5.82 (d, *J* = 10.07 Hz, 1 H) 5.90–5.97 (m, 2 H) 6.46–6.60 (m, 1 H) 6.75–6.80 (m, 2 H) 6.84 (s, 1 H); ^13^C NMR (126 MHz, CHLOROFORM-*d*) δ ppm 16.49, 16.53, 16.99, 27.79, 27.82, 29.98, 30.41, 36.14, 36.19, 38.52, 39.51, 43.30, 45.06, 54.41, 55.20, 69.73, 72.77, 72.84, 78.88, 81.99, 100.94, 104.58, 104.87, 108.04, 108.40, 114.87, 114.92, 121.16, 121.18, 124.04, 125.59, 125.68, 126.71, 132.49, 133.25, 139.04, 147.02, 147.73; HRMS (ESI): calcd. for C_26_H_36_NaO_5_ [M + Na]^+^ 451.2455; found 451.2456.

**26** (74 mg, 74.4% yield). ^1^H NMR (500 MHz, CHLOROFORM-*d*) δ ppm 1.20–1.54 (m, 3 H) 1.57–1.91 (m, 10 H) 1.95–2.17 (m, 5 H) 2.34–2.49 (m, 4 H) 3.32–3.39 (m, 3 H) 3.41–3.51 (m, 2 H) 4.29–4.37 (m, 1 H) 4.42–4.48 (m, 2 H) 4.91–5.02 (m, 2 H) 5.05–5.16 (m, 2 H) 5.82 (d, *J* = 10.53 Hz, 1 H) 6.46–6.59 (m, 1 H) 7.22–7.27 (m, 5 H); ^13^C NMR (126 MHz, CHLOROFORM-*d*) δ ppm 16.02, 16.49, 16.53, 16.59, 16.98, 27.79, 27.82, 29.98, 30.40, 36.13, 36.18, 38.52, 38.54, 39.51, 39.97, 43.29, 45.06, 54.42, 55.20, 69.86, 69.89, 72.46, 72.54, 78.88, 81.99, 104.58, 104.88, 114.89, 114.92, 124.07, 125.59, 125.68, 126.73, 126.75, 128.25, 128.27, 133.25, 135.54, 137.58, 139.01, 139.04, 141.05; HRMS (ESI): calcd. for C_26_H_38_SNaO_3_ [M + Na]^+^ 453.2434; found 453.2436.

#### General procedure for preparation of the alcohols **8**, **13**, **15**, **22**

To a solution of olefin (**9, 24, 14** or **20**) (1 equiv.) in THF (1 ml per 0,3 mmol) cooled to −10 °C was added 0,5 M THF solution of 9-BBN (2 equiv.). The solution was stirred 1 h at 0 °C then excess 9-BBN was carefully quenched with couple drops of water followed by 1 equiv. of 3 M aqueous NaOH and 3 equiv. of 30% aqueous hydrogen peroxide. The reaction mixture was allowed to warm at room temperature with an additional stirring for 1 h. Then the mixture was diluted with water and extracted 3 times with diethyl ether. The combined extracts were washed with brine and dried over sodium sulfate. Removal of the solvent left an oil, which was purified by silica gel chromatography (eluent EtOAc/Hexane 1:10) to give the product **8, 13, 15** or **22** as a colorless oil.

**13** (21 mg, 27% yield). ^1^H NMR (500 MHz, CHLOROFORM-*d*) δ ppm 1.18 (s, 9 H) 1.28–1.55 (m, 2 H) 1.47–1.60 (m, 1 H) 1.52–1.52 (m, 1 H) 1.61 (s, 3 H) 1.62–1.68 (m, 2 H) 1.66–2.02 (m, 1 H) 1.68–1.75 (m, 3 H) 1.92–2.11 (m, 4 H) 2.11–2.44 (m, 1 H) 2.20–2.31 (m, 2 H) 3.29–3.36 (m, 2 H) 3.32–3.38 (m, 3 H) 3.52–3.84 (m, 2 H) 4.34 (td, *J* = 8.96, 4.35 Hz, 1 H) 4.87–5.05 (m, 1 H) 5.15 (m, 1 H); ^13^C NMR (126 MHz, CHLOROFORM-*d*) δ ppm 16.01, 16.13, 16.57, 17.03, 27.58, 28.64, 28.67, 30.03, 30.20, 31.47, 31.50, 36.21, 36.26, 38.46, 38.48, 39.54, 39.84, 43.01, 45.06, 54.37, 55.18, 61.14, 61.19, 62.45, 72.57, 78.90, 81.96, 104.48, 104.85, 120.17, 120.56, 123.82, 126.38, 138.06, 138.33, 139.42, 141.38; HRMS (ESI): calcd. for C_22_H_40_NaO_4_ [M + Na]^+^ 391.2819; found 391.2824.

**22** (40 mg, 44.4% yield). ^1^H NMR (500 MHz, CHLOROFORM-*d*) δ ppm 1.04–1.59 (m, 14 H) 1.63–1.85 (m, 8 H) 1.95–2.06 (m, 2 H) 2.09–2.30 (m, 5 H) 2.35–2.46 (m, 1 H) 3.31–3.38 (m, 3 H) 3.58–3.65 (m, 2 H) 4.01–4.11 (m, 2 H) 4.15–4.36 (m, 1 H) 4.86–5.05 (m, 1 H) 5.10–5.23 (m, 2 H); ^13^C NMR (126 MHz, CHLOROFORM-*d*) δ ppm 16.01, 16.05, 16.12, 16.17, 16.46, 23.47, 23.50, 23.60, 27.22, 27.59, 28.50, 28.84, 30.04, 30.16, 30.23, 31.51, 31.53, 35.81, 35.85, 38.45, 38.48, 38.76, 39.50, 39.77, 39.78, 42.88, 43.06, 44.90, 54.43, 54.45, 55.10, 55.21, 62.46, 62.54, 63.73, 63.98, 64.06, 78.41, 78.79, 81.49, 81.82, 104.46, 104.57, 104.77, 104.90, 120.19, 120.58, 120.62, 120.92, 125.34, 127.15, 127.62, 127.64, 127.73, 128.38, 138.44, 139.27, 140.37, 141.39, 178.55; HRMS (ESI): calcd. for C_23_H_40_NaO_5_ [M + Na]^+^ 419.2768; found 419.2776.

**15** (15 mg, 29% yield). ^1^H NMR (500 MHz, CHLOROFORM-*d*) δ ppm 1.18–1.50 (m, 2 H) 1.50–1.53 (m, 1 H) 1.61 (s, 3 H) 1.70–1.75 (m, 3 H) 1.75–1.84 (m, 2 H) 1.93–2.06 (m, 3 H) 2.08–2.16 (m, 2 H) 2.22–2.30 (m, 2 H) 2.35–2.48 (m, 1 H) 3.29–3.40 (m, 3 H) 3.48 (t, *J* = 6.49 Hz, 2 H) 3.54–3.68 (m, 2 H) 4.33 (t, *J* = 9.00 Hz, 1 H) 4.50 (s, 2 H) 4.88–5.04 (m, 1 H) 5.08–5.23 (m, 2 H) 7.27–7.30 (m, 1 H) 7.30–7.38 (m, 4 H); ^13^C NMR (126 MHz, CHLOROFORM-*d*) δ ppm 16.00, 16.11, 16.56, 17.01, 27.83, 27.88, 30.00, 30.17, 31.49, 31.51, 36.14, 36.18, 38.44, 38.47, 39.51, 39.81, 43.00, 45.04, 54.39, 55.19, 62.47, 70.00, 72.91, 72.98, 78.87, 81.91, 104.51, 104.87, 120.14, 120.56, 124.04, 126.60, 127.53, 127.63, 127.64, 128.38, 138.09, 138.37, 138.57, 139.17, 141.12; HRMS (ESI): calcd. for C_25_H_39_NaO_4_ [M + Na]^+^ 425.2662; found 425.2646.

### The Saturation Transfer Difference (STD) NMR experiment

#### Sample preparation

The deuterated buffer solution was prepared from phosphate buffered saline tablet and D_2_O to get 10 mM phosphate buffer solution. Proteins E6 (HPV type 16) and E7 (HPV type 16) were obtained from MyBiosource (cat# MBS1200699 (E6) and MBS1264011 (E7)) as glycerol containing buffer solutions. The proteins were treated to remove glycerol using the following procedure: The sample was transferred to the amicon column, centrifuged at 12000 rpm for 5 min after which the protein was retained on the column, and the glycerol was removed. Then D_2_O was added to the column and centrifuged again, and this process is repeated 3 times to remove the glycerol completely. After the final wash, required amount of D_2_O was added, the protein was solubilized and taken for the experiment. Ligands **10** and **8** were dissolved in DMSO-d6 to prepare 5 mM stock solutions.

Four NMR samples were prepared from 40 µl of ligand stock solution, 200 µl of protein stock solution, 200 µl of D_2_O buffer solution and 100 µl of DMSO-d6.

#### NMR experiment

The STD-NMR experiment was essentially performed as previously published^13^ using the standard Bruker pulse sequence STDDIFFESGP.3. The experiment was recorded at 25 °C. The protein was saturated at 300 Hz (on resonance), off resonance – 20000 Hz frequency was used. The results are presented in the Supplementary Fig. 2.

### *In silico* analysis

#### Structure preparation

The X-ray structure of full-length human papillomavirus oncoprotein E6 in complex with LxxLL peptide of ubiquitin ligase E6AP (PDB ID: 4GIZ; 2.55 Å resolution)^10^ was retrieved from Protein Data Bank (PDB; http://www.pdb.org/pdb/home/home.do). In order to obtain good information, the protein and the ligand need initial preparation before it was subjected to any *in-silico* analysis. Initially, the maltose binding periplasmic protein and the LxxLL peptide were removed. The resulting E6 protein (chain C) was prepared by following steps. (i) the polar hydrogen atoms and any missing atoms were added to the E6 protein structure by the fully automated Protein preparation wizard^16^ in Maestro 9.6 v suite (Schrödinger, LLC, New York, NY). (ii) all the compounds were prepared for docking studies using LigPrep v2.8, which adds any missing hydrogen atoms, assigns formal charges and generates a set of plausible poses based on ionization and tautomeric states, all of which were converted to 3D representations using the all-atom OPLS2005 force field^17^.

#### Molecular docking

All of the pre-processed ligand derivatives were docked to the hydrophobic binding pocket of E6-HPV16 protein using the automated Induced Fit Docking (IFD)^4^ approach using default parameters: Prime 2.0^18^, Glide 5.0^19^, Extra Precision (XP) mode. The 30 × 30 × 30 Å grid was set up at the center of '*LXXLL* LxxLL hydrophobic binding pocket, where each ligand conformer was centered within this grid. The docked complexes were ranked based on Glide score.

### Cell culture

Cervical cancer cells (SiHa (HPV 16) and HeLa (18)) and the patient derived HPV positive head and neck cancer cells (UT-SCC 60 A, UT-SCC 60B, UT-SCC 65, and UT-SCC 69) were cultured in DMEM (Sigma-Aldrich, St Louis, MO, USA) supplemented with 10% fetal calf serum (BioClear, Wiltshire, UK), 100 U/ml penciliin, 2 mM L-glutamin, and 100 µg/ml streptomycin (Sigma-Aldrich). The primary skin fibroblast cell lines, K87 and K74, were cultured in MEM media (Sigma-Aldrich) supplemented with 15% fetal calf serum (BioClear, Wiltshire, UK), 100 U/ml penciliin, 2 mM L-glutamin, and 100 µg/ml streptomycin (Sigma-Aldrich).

### Apoptosis parameters

SiHa cells were treated with 0–40 µM compounds **8–10** and **13–26**. After 24 hrs, the cells were collected and assayed for apoptosis. Activated caspase-3 in SiHa cells was labeled with the phyco-erythrin-conjugated antibody in accordance with the manufacturer’s protocol (PE Active Caspase-3 Apoptosis Kit; BD Pharmingen, San Diego, CA) and analyzed by FACSCalibur flow cytometer (FL-2, FSC, BD Pharmingen). The assessment of apoptosis by flow cytometric technique was performed on SiHa cells that were plated in a 96-well plate as triplicate samples. The cancer cells were pretreated with various concentrations of compounds **8** and **10** for 0–6 hour after which the plate was centrifuged with a culture plate rotor (1000 rpm, 5 minutes). For analysis of nuclear fragmentation, propidium iodide (PI) buffer (0.3% Triton X-100, 40 mM Na-citrate, and 50 µg/ml PI) (Sigma) was added to the well plate. After 10 minutes of incubation period at room temperature, the plate was analyzed with LSRII flow cytometer equipped with HTS platform (PE-A channel). The fraction of sub-G0/G1 events was rated as a measure of apoptotic cell death.

### Cell cycle analysis

Cells were synchronized by serum-starvation (1% serum) for 48 h and then cultured in medium containing 10% FCS for 24 h. After this, the cells were incubated with 0 and 20 μM of compounds **8** and **10** for 24 h. The cells were then collected and disrupted and the nuclei labeled for DNA content with PI by re-suspension in sodium citrate buffer (40 mM Na-citrate, 0.3% Triton X-100, 50 μg/ml PI (Sigma–Aldrich)). After incubation at room temperature for 10 min, the samples were immediately analyzed by FACSCalibur flow cytometry.

### *In vitro* p53 degradation assay

The plasmids used in the* in vitro* translation assay, full length, p2207 pGEM p53 was a gift from peter Howley (Addgene plasmid # 1-853)^20^ and human papillomavirus type 16 E6 (MBP-E6)^10^, was a gift from Gilles Trave. Each protein was translated in separate reactions using the protocol provided in the TNT T7 coupled rabbit reticulocyte lysate systems (Promega). For p53 degradation assay^21^, the translation reactions were combined in the absence or presence of the respective compound 8 and 10 at the indicated concentrations or a DMSO control. Following incubation, reactions were analyzed on SDS-PAGE followed by p53 Western blotting.

### Western blotting

The cell lysates were prepared by lysing floating and attached cells in Laemmli sample buffer and boiling the samples for 10 minutes. Proteins were separated by SDS-PAGE and then transferred onto PVDF membrane (Millipore). Western blotting was done using antibodies against HPV E6 (Arbor Vita Corporation), HPV16 E7 (8C9, Invitrogen), p53 (D0-1, Santa Cruz), p21, MBP, poly (ADP-ribose) polymerase (PARP) (46D11, Cell Signaling Technology), β-actin (AC-40, Sigma–Aldrich) and Hsc70 (Enzo Lifesciences). The HRP-conjugated secondary antibodies were from Southern Biotech. The membranes were developed using the ECL method either on X-ray Film (Santa Cruz) or using the iBright FL1000 (Thermo Fisher Scientific).

### *In ovo* chick chorioallantoic membrane (CAM) model

The *in ovo* CAM assay was carried out as described in our earlier report^7^. Concisely, the fertilized chicken white eggs (LSK Poultry Oy, Finland) were incubated for 8 days at 37 °C with about 65% relative humidity. After that, the egg shell was drilled and extended to approximately 3 cm and a polyethylene ring was placed on the top of the allantoic membrane. 1.5 million SiHa cells (with Matrigel, BD Pharmingen) in serum free media (DMEM) were then implemented inside the ring and egg was returned to the incubator. On day 11, various doses of compound (0–6 mg/kg) were added topically to the developed CAM tumor. The treatment was repeated until day 14. The tumor samples were then harvested from the membrane.

### Morphologic staining and immunohistochemistry

Five-micrometer-thick sections were taken perpendicularly from paraffin-fixed CAM tumors samples and processed for HandE staining. All the images were taken with a digital camera (DC300F) attached to a DMLB microscope (Leica). For immunohistochemistry examination of tissue sections, upon antigen revealing, tissues were immunohistochemically stained with HRP conjugated antibody and then visualized by using ABC staining system (Vector lab, CA, USA). Bad antibody (Abcam) was used for immunohistochemistry. At the end, all sections were counter-stained with Mayers hematoxyline (Histolab, Gothenburg, Sweden). Isotype and concentration matched primary antibodies were used as negative controls, and all were found to be negative.

### Statistical analysis

The graphs were prepared with GraphPad Prism software and they represent mean values ± standard error of mean (SEM). The number of independent experiments and the statistical significance is indicated in the figure legends. Statistical significance was determined using Student’s t-test.

## Supplementary information

Supplementary information
