# Supplementary material for: Synthesis and Evaluation of Anisomelic acid-like Compounds for the Treatment of HPV-Mediated Carcinomas
Source: Sci Rep. 2019 Dec 30;9:20295. doi: 10.1038/s41598-019-56410-1 (PMC6937315; doi:10.1038/s41598-019-56410-1)
Supplement: Supplementary file 1 — Supplementary information [file 41598_2019_56410_MOESM1_ESM.docx]

***Supplementary information**

**Synthesis and Evaluation of Anisomelic acid-like Compounds for the Treatment of HPV-Mediated Carcinomas**

**Rajendran Senthilkumar**^1,2,3^**, Yury Brusentsev**^1^**, Preethy Paul**^2,3^**, Parthiban Marimuthu**^4^**, Fang Cheng**^2,5^**, Patrik C. Eklund**^1*^**, John Elias Eriksson**^2,3^

^1^Johan Gadolin Process Chemistry Centre, Åbo Akademi University, c/o Laboratory of Organic Chemistry, Biskopsgatan 8, 20500 Turku, Finland.

^2^Cell Biology, Faculty of Science and Engineering, Åbo Akademi University, Tykistökatu 6A, 20520 Turku, Finland.

^3^Turku Centre for Biotechnology, Åbo Akademi University and University of Turku, Tykistökatu 6A, 20520, Turku, Finland.

^4^Structural Bioinformatics Laboratory, Biochemistry, Faculty of Science and Engineering, Åbo Akademi University , Tykistökatu 6A, 20520 Turku , Finland

^5^School of pharmaceutical sciences (Shenzhen), Sun Yat-sen University, 518107, Shenzhen, China

Correspondence and request for materials should be addressed to P.E.* (email: [paeklund@abo.fi](mailto:paeklund@abo.fi)) or J.E. (email: [jeriksso@abo.fi](mailto:jeriksso@abo.fi)) or R.S. (email: [srajendr@abo.fi](mailto:srajendr@abo.fi))

**Supplementary Table 1.** In silico docking analysis of **8-10** and **13-26** with E6 protein.

| **Comp.No** | **Docking score (kcal/mol)** | **Number of**  **H-bonds** | **Number of salt bridges** | **Bond length** | **Interacting residues** |
| --- | --- | --- | --- | --- | --- |
| **13** | -60.45 | 6 | 1 | 2.8 | Y32,F45,L50,C51,V53,V62,L67,S71,S74,Q107,R129,R131 |
| **14** | -65.57 | 2 | 1 | 2.8 | V31,L50,C51,V53,Y60,A61,V62,S71,S74,E75,I104,Q107,R129,R131 |
| **15** | -58.61 | 4 | 1 | 3.0 | V31,L50,C51,V53,Y60,A61,V62,S71,S74,I104,Q107,R129,R131 |
| **16** | -60.41 | 5 | - | 3.2 | Y32,D49,L50,C51,V62,L67,Y70,S71,S74,R102,Q107,I128,R129,R131,T133 |
| **17** | -44.82 | 3 | 1 | 3.1 | V31,Y32,L50,C51,V53,S71,S74,E75,I104,Q107,I128,R129,R131,T133 |
| **18** | -49.18 | 3 | 1 | 3.3 | V31,F45,C51,V53,V62,Y70,S71,S74,R102,Q107,I128,R129,R131,T133 |
| **19** | -48.04 | 2 | - | 2.8 | V31,Y32,F45,L50,C51,V53,Y60,A61,V62,L67,Y70,S71,S74,Q107,R129,R131,T133 |
| **20** | -63.60 | 3 | 1 | 3.1 | F45,D49,C51,V53,L67,S71,S74,E75,R102,I104,Q107,R129,R131 |
| **21** | -38.18 | 5 | - | 3.2 | F45,C51,V62,L67,Y70,S71,S74,E75,I104,Q107,R131,T133 |
| **22** | -60.33 | 7 | - | 3.1 | V31,Y32,D49,C51,V53,A61,L67,S71,C103,I104,Q107,R131,T133 |
| **23** | -50.31 | 2 | - | 2.9 | V31,L50,C51,V53,A61,V62,L67,S71,S74,E75,Q107,I128,R129,R131,T133 |
| **24** | -28.8 | 3 | 1 | 3.3 | L50,C51,V53,Y60,A61,L67,S71,S74,Q107,R129,R131 |
| **25** | -41.05 | 2 | 1 | 3.0 | V31,F45,L50,C51,V53,A61,V62,L67,Y70,S71,S74,E75,Q107,I128,R129,R131,T133 |
| **26** | -48.78 | 1 | 1 | 3.3 | L50,C51,V53,V62,L67,Y70,S71,S74,Q107,R129,R131,T133 |
| **10** | -63.66 | 2 | 1 | 3.2 | V31,Y32,C51,V53,Y60,A61,L67,S74,I104,Q107,I128,R131,T133 |
| **9** | -60.65 | 4 | - | 3.3 | V31,Y32,C51,V53,Y60,A61,V62,L67,S71,S74,Q107,I128,R129 |
| **8** | -65.74 | 5 | 1 | 3.6 | V31,Y32,C51,I52,V53,Y60,A61,V62,S74,I104,Q107,I128,R129,R131 |

**Suppl. Table 2.**

| **Sample** | **5mM ligand DMSO-d6 solution** | **50 µM protein D_2_O buffer solution** | **D_2_O buffer solution** | **DMSO-d6, µl** |
| --- | --- | --- | --- | --- |
| 1 | **10** (40 µl) | E6 (200 µl) | 200 µl | 100 µl |
| 2 | **10** (40 µl) | E7 (200 µl) | 200 µl | 100 µl |
| 3 | **8** (40 µl) | E6 (200 µl) | 200 µl | 100 µl |
| 4 | **8** (40 µl) | E7 (200 µl) | 200 µl | 100 µl |

**Supplementary Figure 1**

**E6- 8**

**E6- 10**


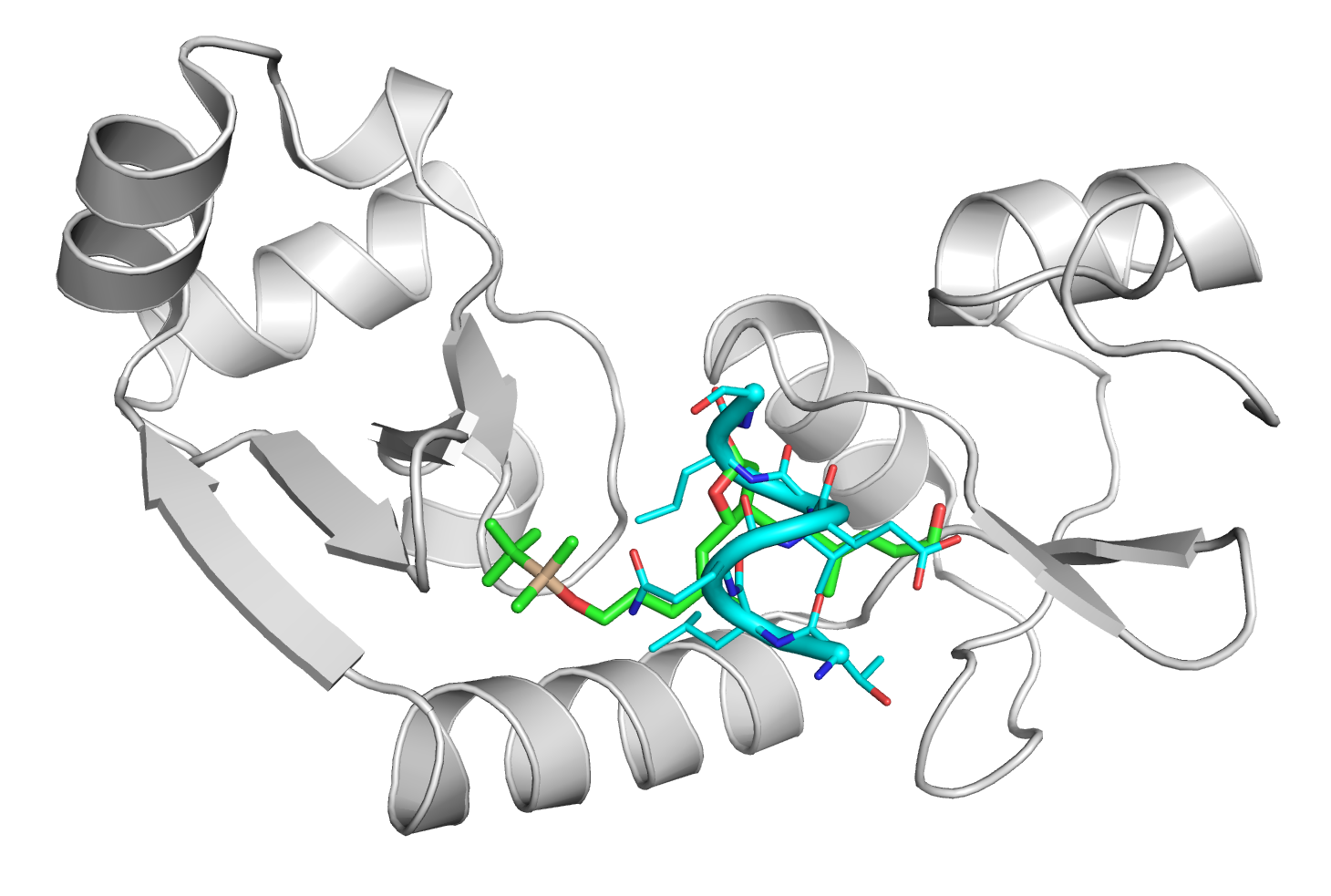

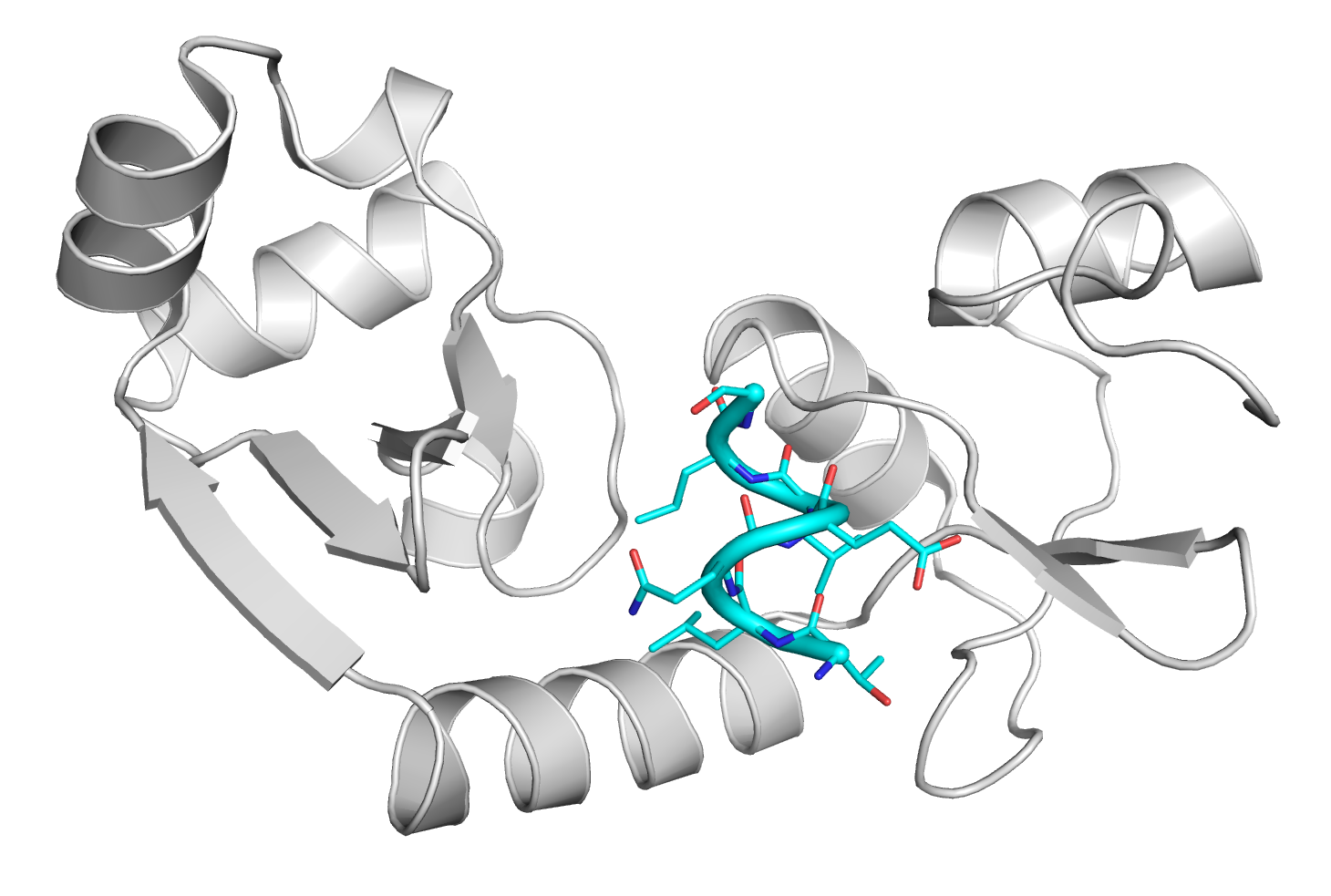


**LxxLL motif**

**LxxLL motif**

**Suppl. Figure 1**. Binding mode of **10** and **8** with E6 protein. **10** and **8** are located deep in the hydrophobic druggable pocket ‘LxxLL’ (hydrophobic cavity) motif.

**Supplementary Figure 2**


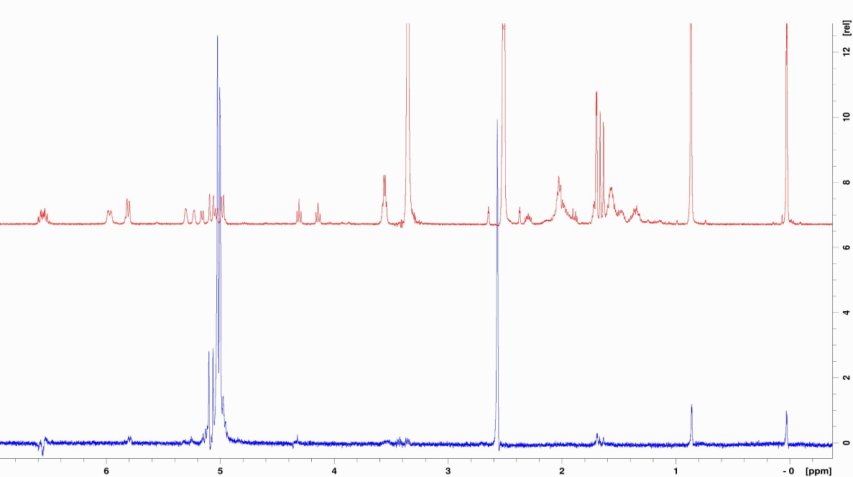


**TBS**

**Methyl groups**

**TBS**

**14**

**DMSO**

**15 &19**

**13**

**5**

**6**

**3**

H^1^ NMR of **10**

STD NMR of E6-**10**


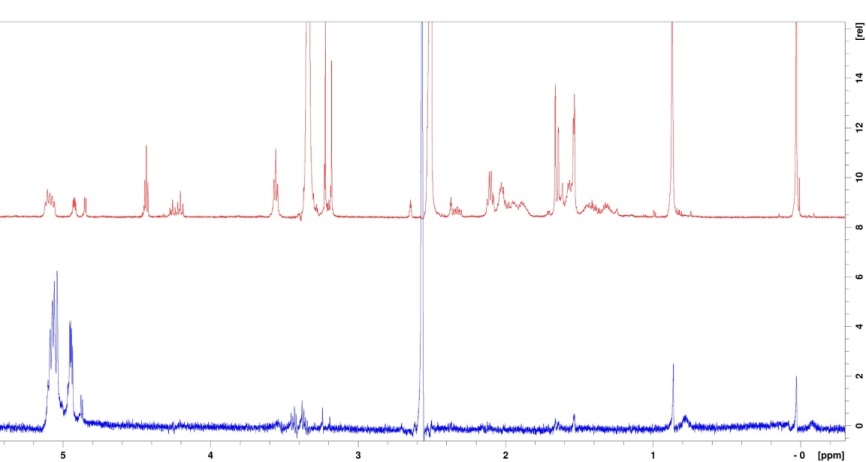


**TBS**

**TBS**

**Methyl groups**

**13, 5**

**& 19**

**22**

**15**

**14**

**3**

**6**

**DMSO**

H^1^ NMR of **8**

STD NMR of E6-**8**


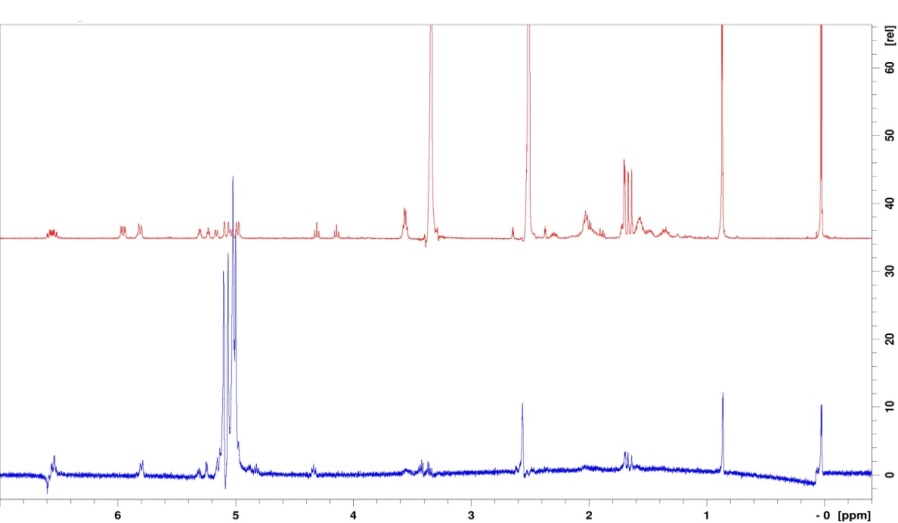


**TBS**

**TBS**

**Methyl groups**

**14**

**DMSO**

**15 &19**

**13**

**5**

**6**

**3**

H^1^ NMR of **10**

STD NMR of E7-**10**


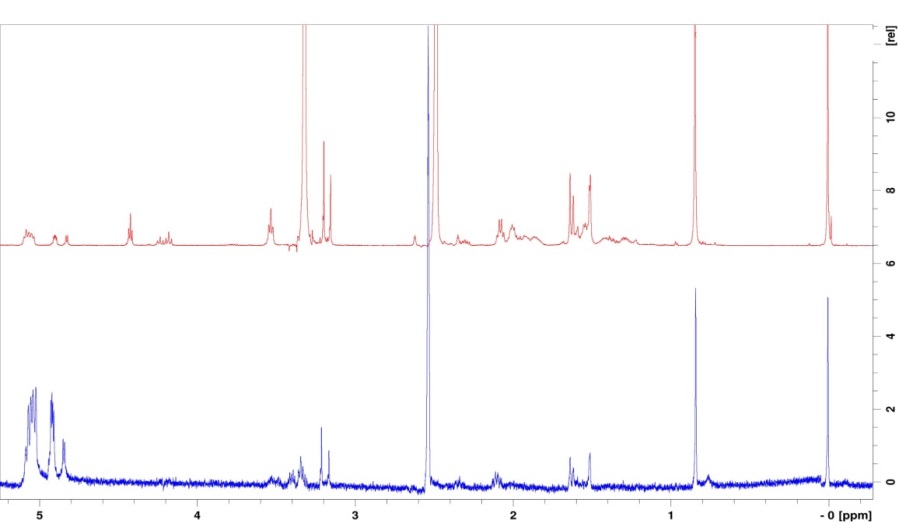


**TBS**

**TBS**

**Methyl**

**13, 5**

**& 19**

**22**

**15**

**14**

**3**

**6**

**DMSO**

H^1^ NMR of **8**

STD NMR of E7-**8**

**Suppl. Figure 2**. STD-NMR experiments. The blue spectrum shows the enhancement of signal intensity (for compounds 8 and 10) after substraction of the reference spectra (pure compounds, red). The enhancement by saturation-transfer is especially observed for the vinyl protons of **10** or **8**. But clear interactions can also be seen at the TBS group, methyl groups and at the unsaturated moieties, indicating that the binding was mostly caused by hydrophobic interactions.

**Supplementary Figure 3**


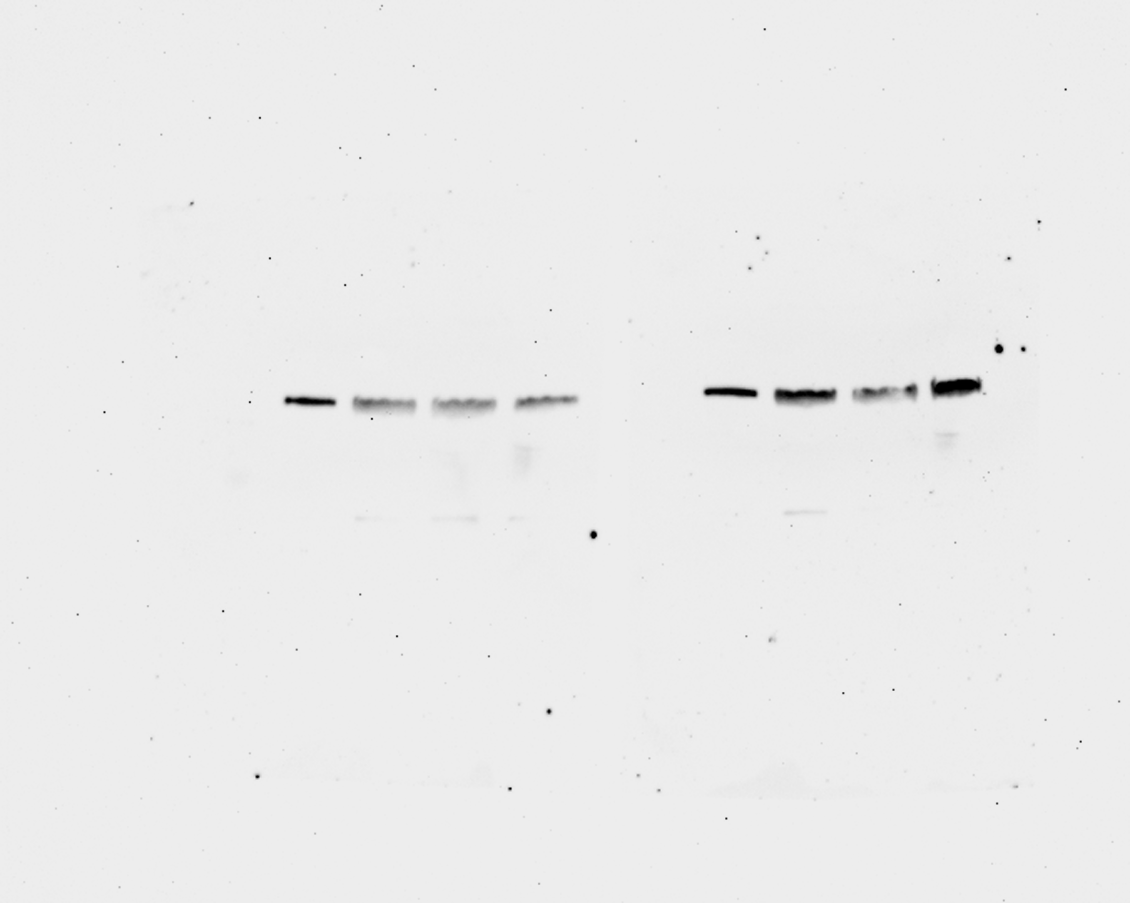


6h (40µM)

C **10** **8**

(E)

(B)

(A)

(C)


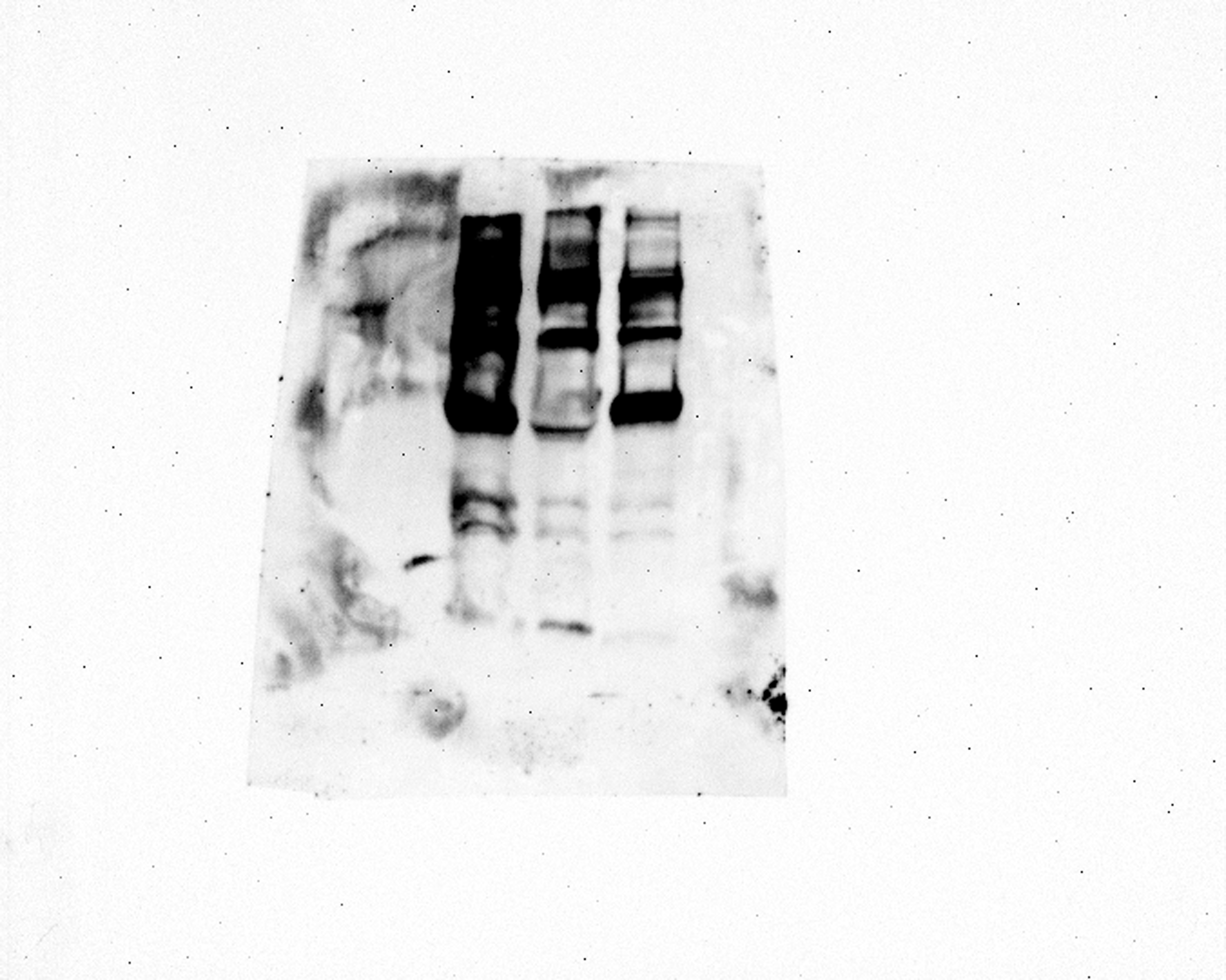


C **10** **8**

6h (40µM)


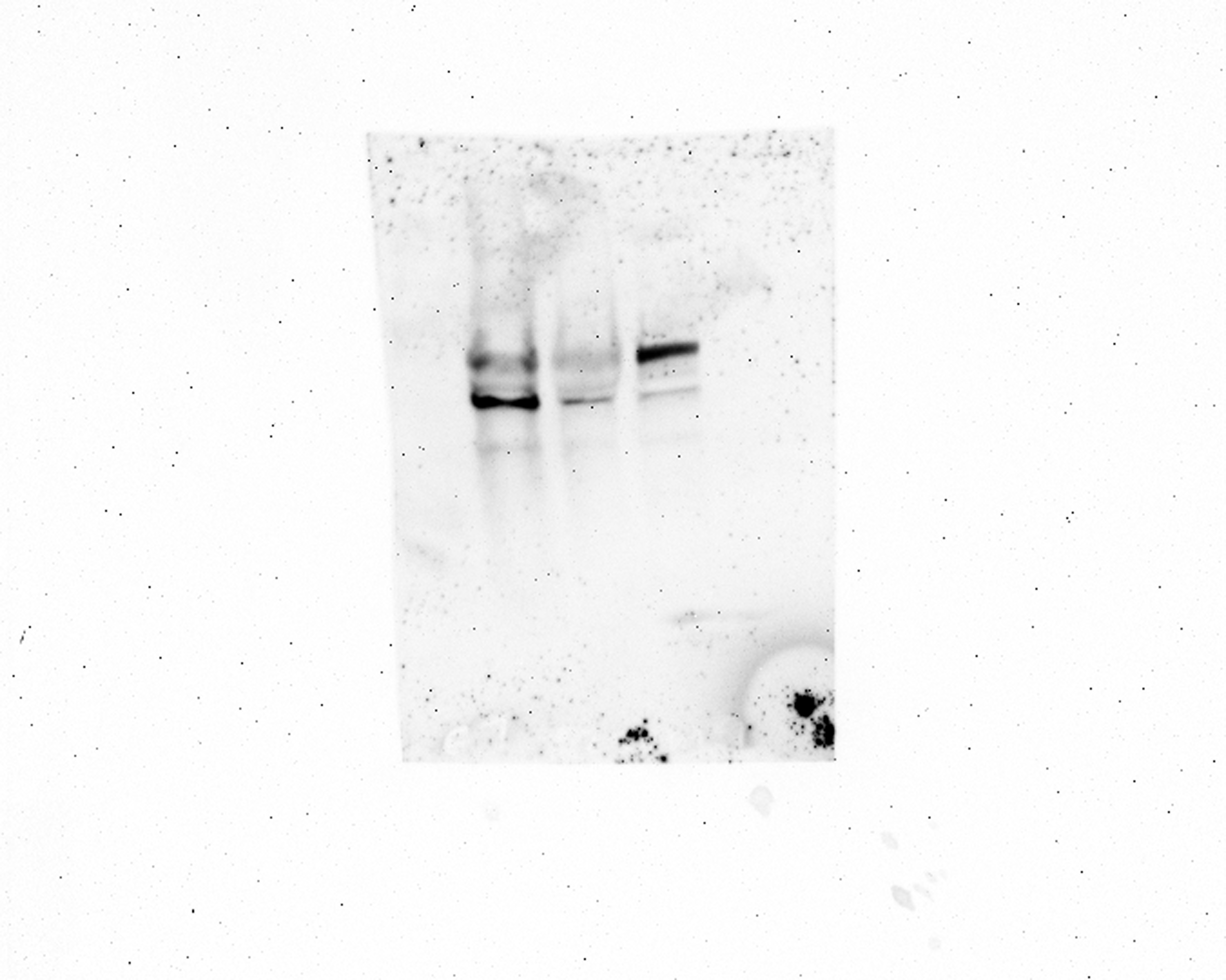


C **10** **8**

High exposure

Low exposure

6h (40µM)

(D)

24h (40µM)

24h (40µM)


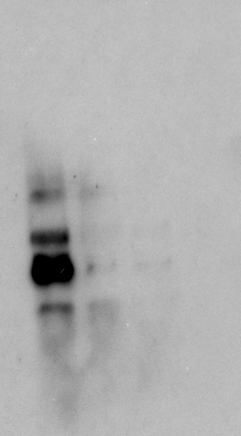


6h (40µM)

C **10** **8**

C **10** **8**

C **10** **8**


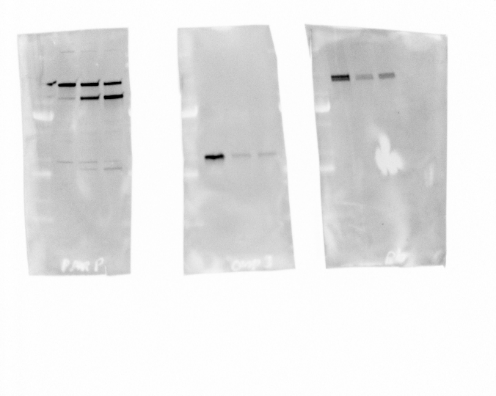

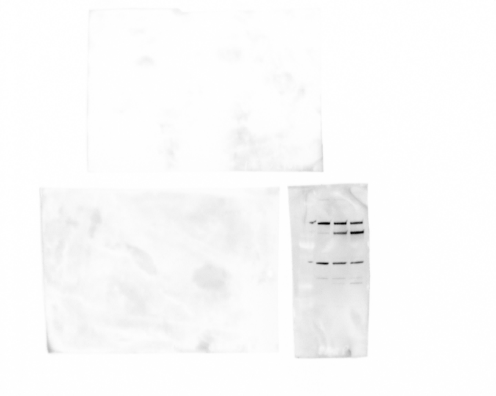


HPV E6

Hsc70

HPV E7

β-Actin

PARP


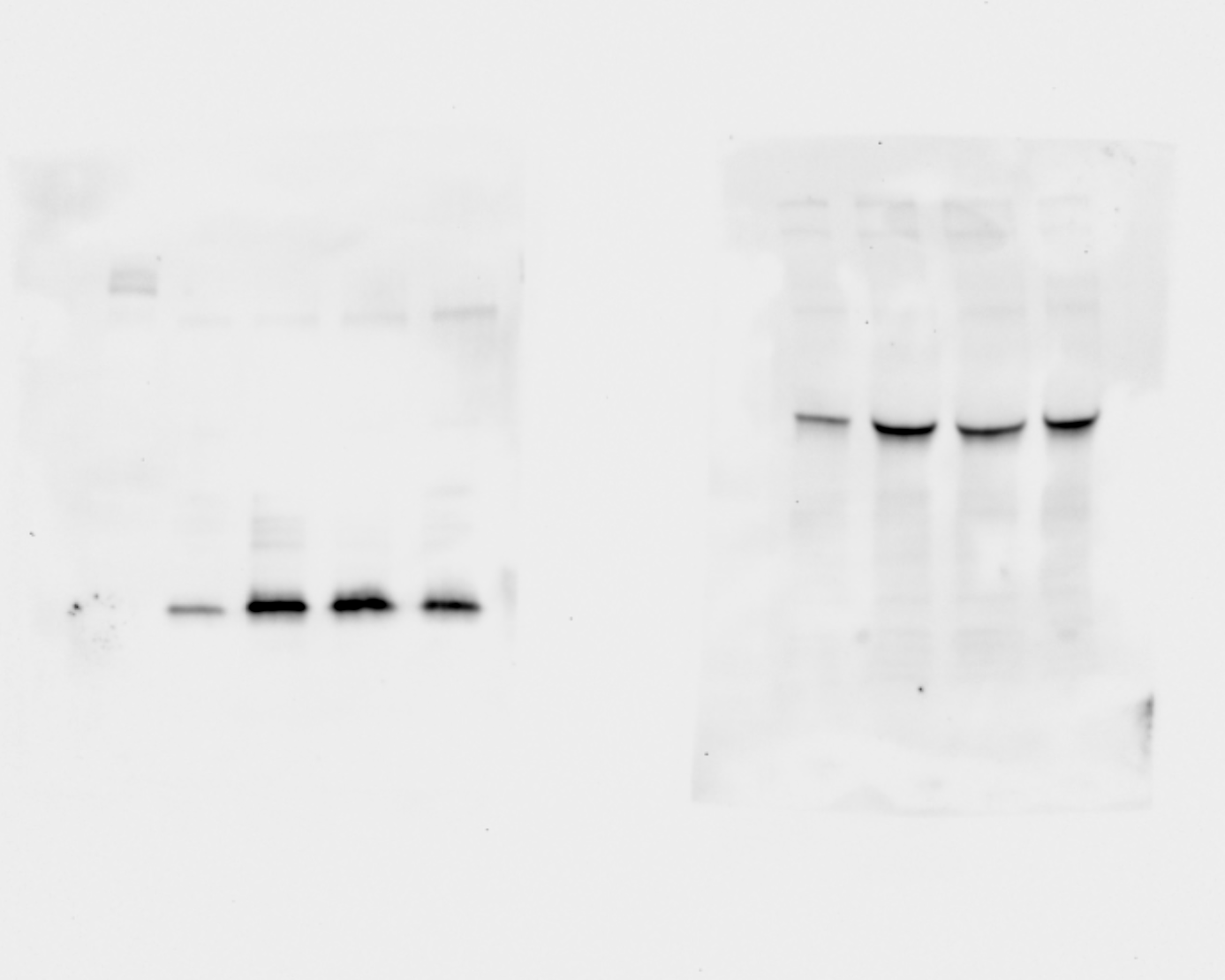


24h (20µM)

C **10** **8**


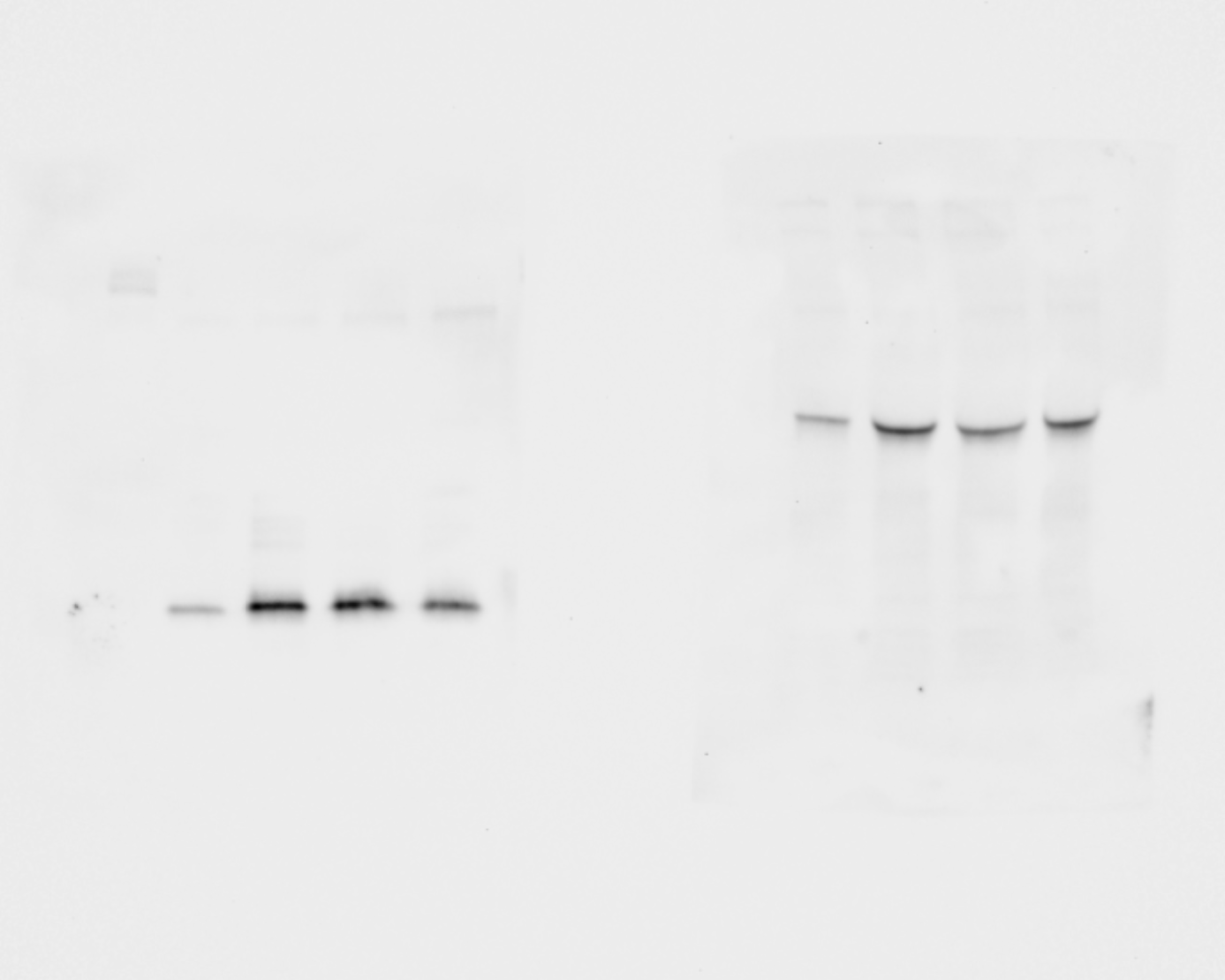


24h (20µM)

C **10** **8**


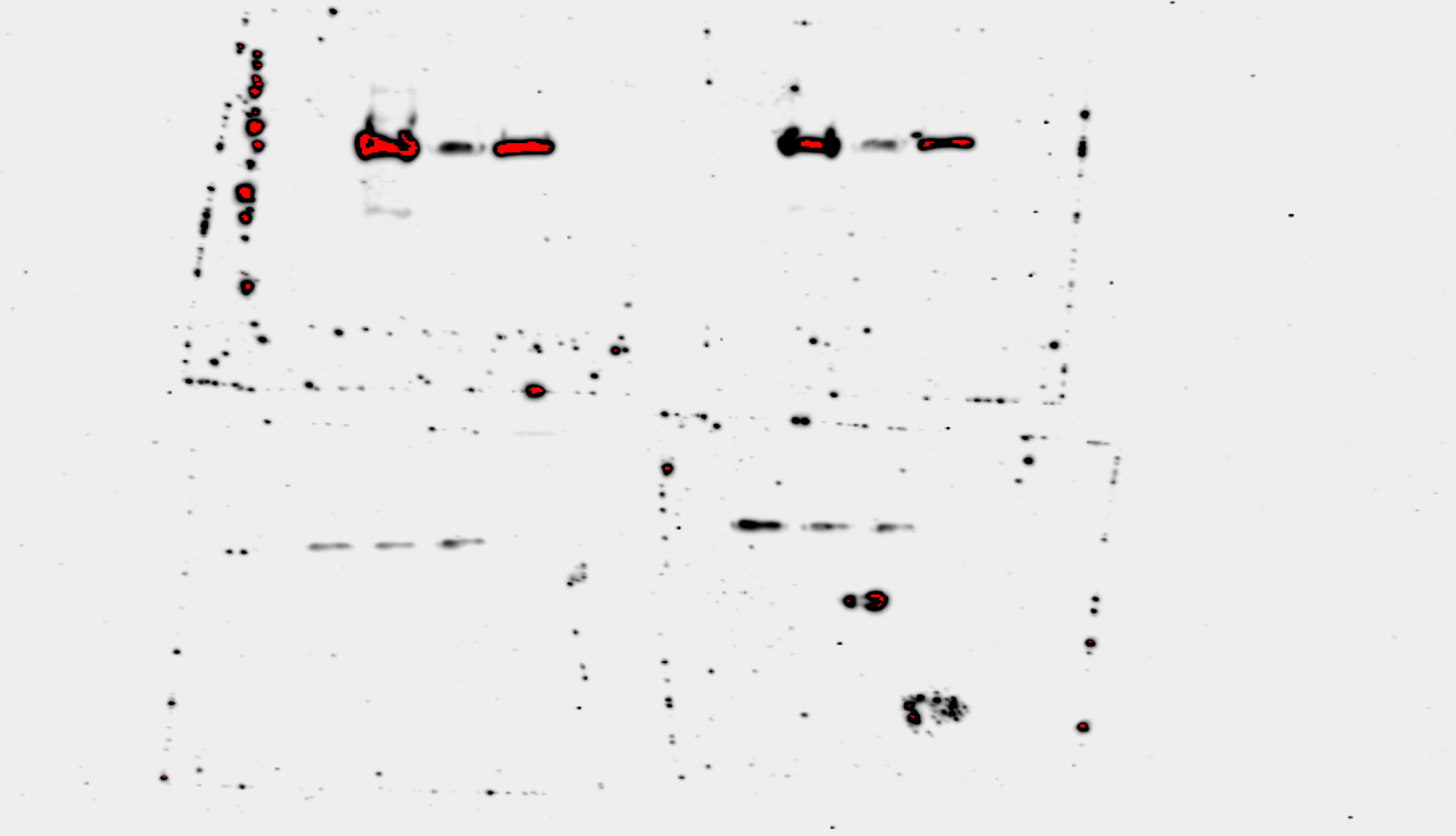


24h (20µM)

C **10** **8**

(F)

(G)

(H)

Hsc70

p21

p53

(I)


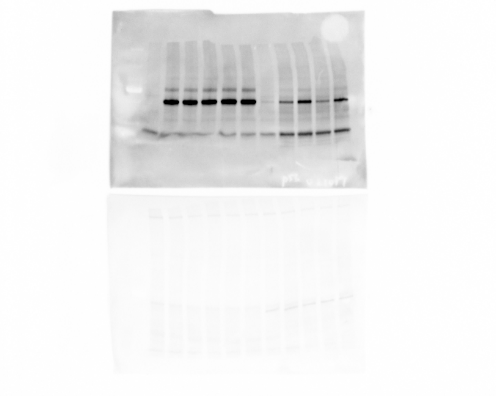

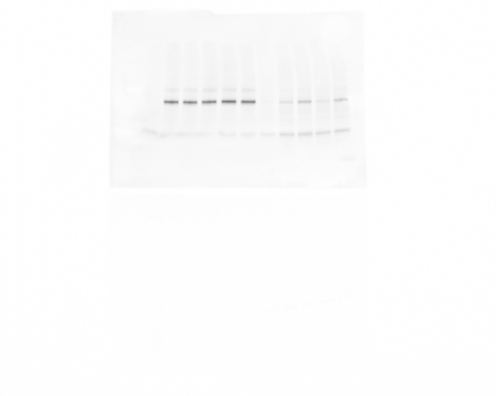


Low exposure

High exposure

p53

p53

(J)

(K)


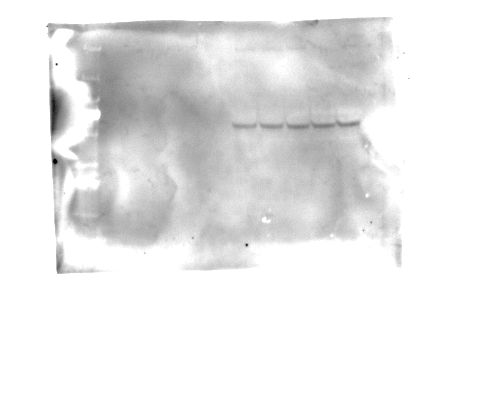

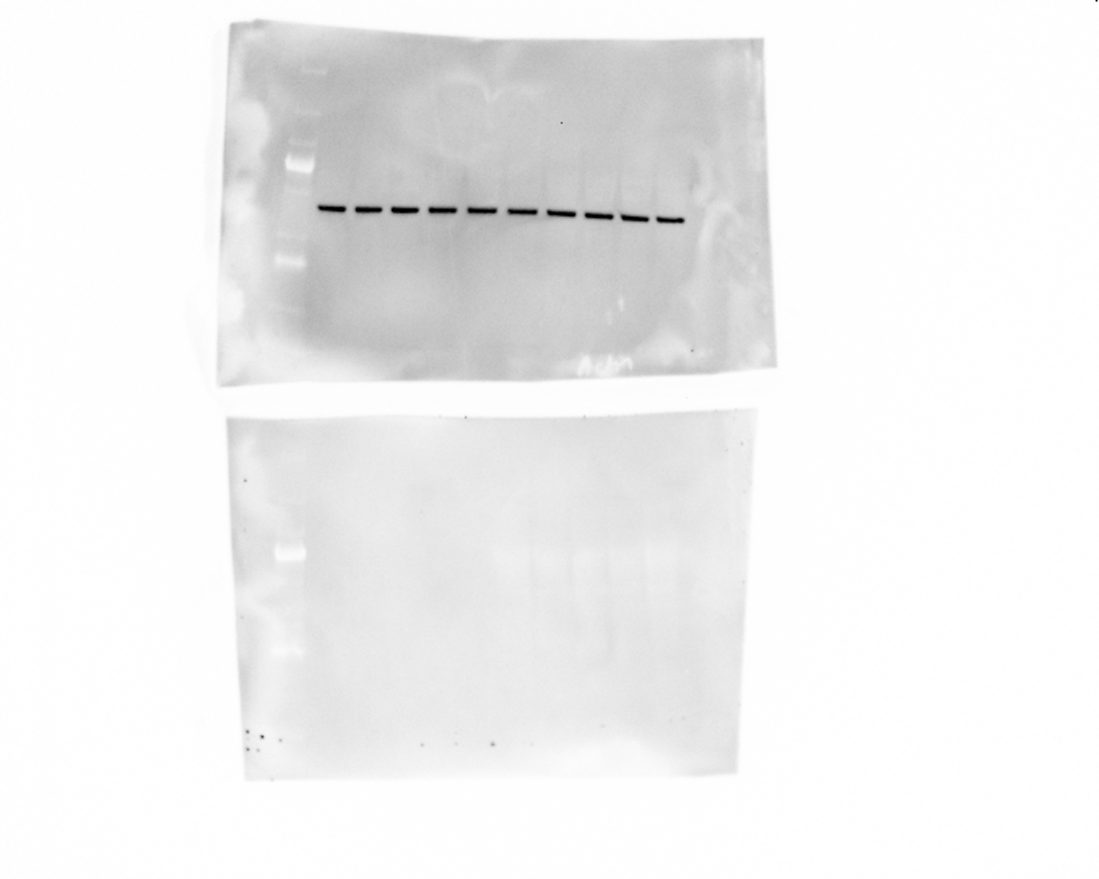


MBP

β-Actin

**Supplementary Figure 3**. The full-length blots of the cleavage of PARP (116/85) (A), downregulation of HPV-E6 (C), HPV-E7 (D) oncoproteins and upregulation of p53 (F) and p21 (G). (B) & (K) are β-Actin loading control, (E) & (H) are Hsc70 loading control blots. Full-length blot of p53 (I) & MBP (J) obtained from the p53 degradation assay.
